# Rare Ophiuroid-Type Steroid 3β,21-, 3β,22-, and 3α,22-Disulfates from the Slime Sea Star *Pteraster marsippus* and Their Colony-Inhibiting Effects against Human Breast Cancer Cells

**DOI:** 10.3390/md22010043

**Published:** 2024-01-12

**Authors:** Alla A. Kicha, Timofey V. Malyarenko, Alexandra S. Kuzmich, Olesya S. Malyarenko, Anatoly I. Kalinovsky, Roman S. Popov, Dmitriy K. Tolkanov, Natalia V. Ivanchina

**Affiliations:** 1 G.B. Elyakov Pacific Institute of Bioorganic Chemistry, Far Eastern Branch, Russian Academy of Sciences, Pr. 100-let Vladivostoku 159, 690022 Vladivostok, Russia; malyarenko-tv@mail.ru (T.V.M.); assavina@mail.ru (A.S.K.); malyarenko.os@gmail.com (O.S.M.); kaaniw@piboc.dvo.ru (A.I.K.); prs_90@mail.ru (R.S.P.); tolkanov.dk@gmail.com (D.K.T.); 2 Department of Bioorganic Chemistry and Biotechnology, School of Natural Sciences, Far Eastern Federal University, Russky Island, Ajax Bay, 10, 690922 Vladivostok, Russia

**Keywords:** steroid, disulfate, tryptamine, spectra NMR, starfish, *Pteraster marsippus*, cytotoxic activity, colony-inhibiting activity

## Abstract

Two new steroid 3β,21-disulfates (**1**, **2**) and two new steroid 3β,22- and 3α,22-disulfates (**3**, **4**), along with the previously known monoamine alkaloid tryptamine (**5**) were found in the ethanolic extract of the Far Eastern slime sea star *Pteraster marsippus*. Their structures were determined on the basis of detailed analysis of one-dimensional and two-dimensional NMR, HRESIMS, and HRESIMS/MS data. Compounds **1** and **2** have a Δ^22^-21-sulfoxy-24-norcholestane side chain. Compounds **3** and **4** contain a Δ^24(28)^-22-sulfoxy-24-methylcholestane side chain, which was first discovered in the polar steroids of starfish and brittle stars. The influence of substances **1**–**4** on cell viability, colony formation, and growth of human breast cancer T-47D, MCF-7, and MDA-MB-231 cells was investigated. It was shown that compounds **1** and **2** possess significant colony-inhibiting activity against T-47D cells, while compounds **3** and **4** were more effective against MDA-MB-231 cells.

## 1. Introduction

Highly oxidized steroid compounds are low-molecular-weight metabolites of sea stars (starfish) that belong to the phylum Echinodermata, class Asteroidea. These polar substances differ from related ones from other marine invertebrates in terms of their significant structural diversity and are subdivided into several structural groups, including polyhydroxysteroids, mono-, di-, and triglycosides of polyhydroxysteroids, steroid alkaloids, steroid conjugates with fatty acids, cyclic steroid triglycosides, and asterosaponins—steroid glycosides with carbohydrate moieties from three to six monosaccharide residues [1,2,3,4]. Polar steroid compounds derived from sea stars were shown to exhibit a considerable variety of physiological effects, namely antimicrobial, antiviral, immunomodulatory, neurotrophic, anti-inflammatory, and others [5,6,7,8]. In the last two decades, many new reports have appeared on studies of the antitumor activity of polar steroid compounds from starfish as well as on possible molecular mechanisms of action of these compounds [9,10].

Starfish steroids often contain a single sulfate group, which can be located either in the tetracyclic core or side chain, or in the carbohydrate chain of molecule. In contrast to starfish, representatives of another class of echinoderms—ophiuroids or brittle stars (Ophiuroidea)—contain the characteristic steroid 3α,21-disulfates with mainly 5β- or Δ^5^-cholestane cores, which are distinctive features of these animals [11,12,13,14]. Notably, such steroid 3β(or α),21-disulfates have been found in six starfish species of the Pterasteridae family instead of polar steroid metabolites common in representatives of the class Asteroidea [15]. The physiological properties of ophiuroid-like steroid compounds from the sea stars of the Pterasteridae family have been insufficiently studied so far. Previously, these compounds from the starfish *P. pulvillus* were only shown to exhibit hemolytic activity on mouse erythrocytes [16]. Recently, we have investigated the structures of new steroid 3β,21- and 3β,22-disulfates from the sea star *Pteraster marsippus* that was collected off the coast of the Kuril Islands (Sea of Okhotsk) [15]. The mixture of two substances with oxo-group at the C-7 position in the tetracyclic pattern of *P. marsippus* was determined to exhibit a significant cytotoxic effect against two- and three-dimensional cultures of human breast carcinoma ZR-75-1 cells [15]. In the present paper, in continuation of our investigation on the fraction of disulfated steroids from *P. marsippus*, the isolation and characterization of new disulfate ophiuroid-type steroid compounds **1**–**4** and the known compound **5** are described. Moreover, the cytotoxic activity and capability of compounds **1**–**4** to inhibit the viability, colony formation and growth of human breast cancer T-47D, MCF-7, and MDA-MB-231 cells are reported. We did not use normal cells, since the amount of isolated substances was limited (did not exceed 1 mg) and previous work showed that substances of this structural group did not exhibit significant cytotoxicity against human epithelial kidney HEK293 cells at concentrations of up to 100 μM [15].

## 2. Results and Discussion

### 2.1. Structure Determination of Compounds **1**–**5**

**Figure 1:**
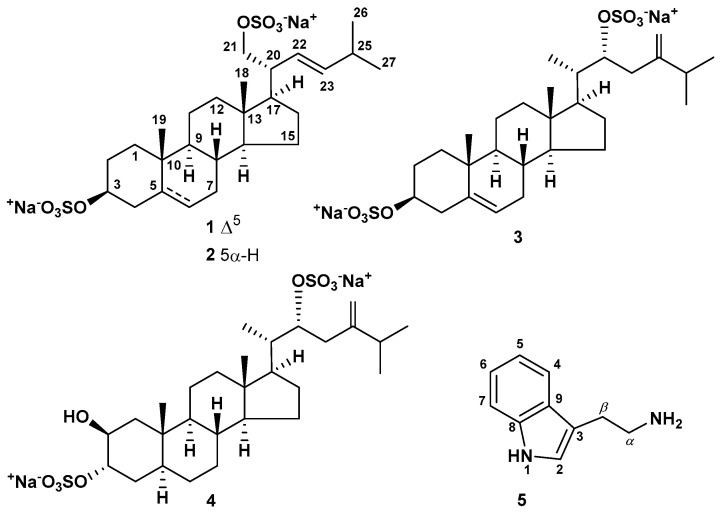
Chemical structures of steroids **1**−**4** and compound **5**.

The concentrated ethanol extract of the sliced specimens of the sea star *P. marsippus* was chromatographed on a column with Polychrome 1. The total fraction of steroid compounds was eluted with 50% aq. EtOH and separated by sequential chromatography on Si gel and Florisil columns using CHCl_3_/EtOH/H_2_O (stepwise gradient) eluent systems to afford 11 fractions. HPLC of fractions 8 and 9 on Discovery C18 (10.0 × 250 mm), YMC-Pack Pro C18 (10.0 × 250 mm), and YMC-Pack Pro C18 (4.6 × 250 mm) columns gave new disulfated steroid compounds **1**–**4** and the known monoamine alkaloid tryptamine (**5**) (Figure 1).

**Figure 2:**
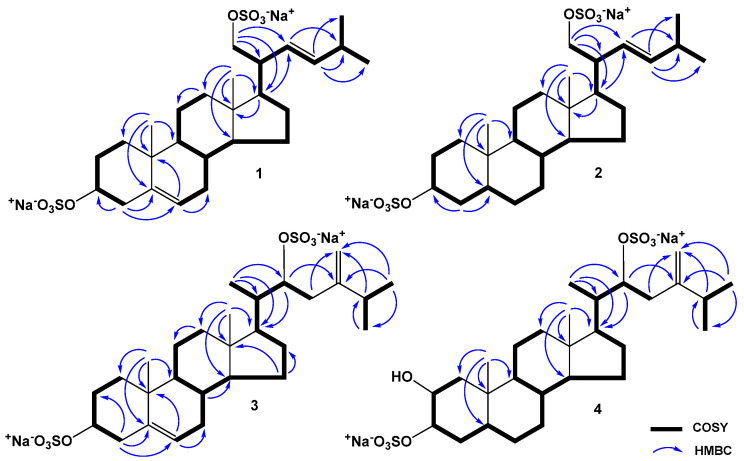
^1^H-^1^H COSY and main HMBC correlations of steroids **1**–**4**.

**Figure 3:**
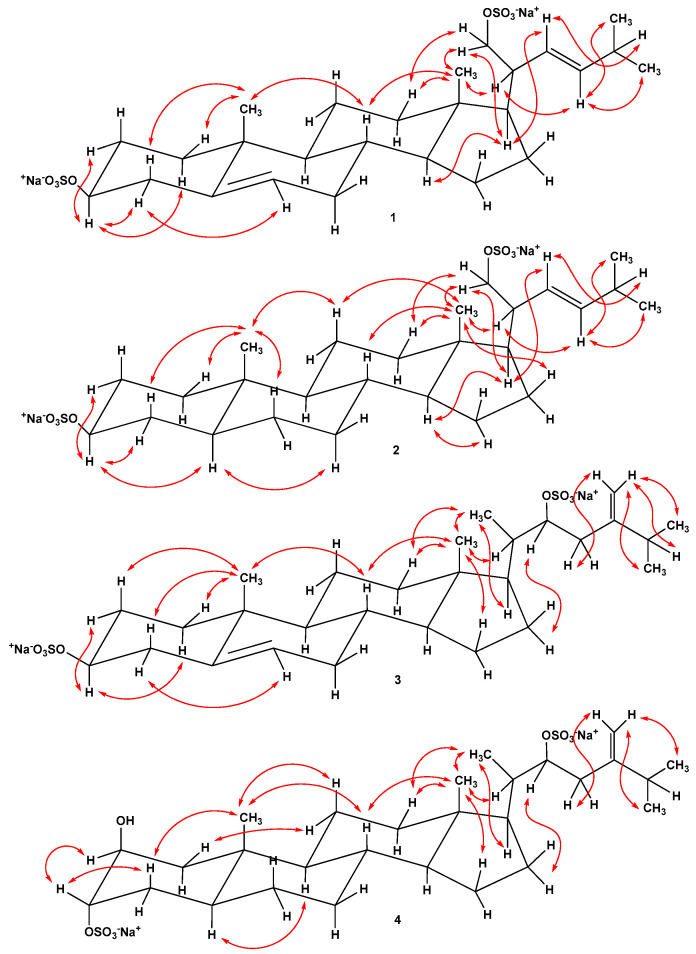
Main ROESY correlations for steroids **1**–**4**.

Steroid **1** had the molecular formula C_26_H_40_O_8_S_2_Na_2_, determined from the peaks of [M − Na]^−^ ion at *m*/*z* 567.2073 and [M − 2Na]^2−^ ion at *m*/*z* 272.1090 in the (−)HRESIMS, and from the peak of cationized molecule [M + Na]^+^ at *m*/*z* 613.1847 in the (+)HRESIMS. The HRESIMS, NMR data and the presence in the (−)HRESIMS/MS spectrum of precursor ion [M − 2Na]^2−^ at *m/z* 272.1076 of peaks of fragment ions at *m/z* 447.2561 [M − 2Na − HSO_4_]^−^ and 96.9611 [HSO_4_]^−^ corresponded to a C_26_ disulfated steroid diol with two degrees of unsaturation (Figure S1). The signals of protons and carbons of angular methyl groups (*δ*_H_ 0.75 s, *δ*_C_ 12.9, CH_3_-18) and (*δ*_H_ 1.03 s, *δ*_C_ 19.7, CH_3_-19), an oxidized methine (*δ*_H_ 4.13 m, *δ*_C_ 79.9, CH-3), and the Δ^5^ double bond (*δ*_H_ 5.38 m; *δ*_C_ 141.6, 123.2) were present in the ^1^H- and ^13^C-NMR spectra of **1** (Table 1 and Table 2, Figures S2 and S3). The resonanses of protons and carbons of CH-3, C-5, CH-6, CH_3_-18, and CH_3_-19 and the broad multiplet of H-3 (1/2 Δ*W* = 19.8 Hz) showed the Δ^5^-3β-sulfoxy tetracyclic moiety in **1**, like it was observed in the related (20*R*)-24-methylcholesta-5,24(28)-diene-3β,21-diol 3β,21-disulfate previously obtained from *P. marsippus* [15]. The ^1^H-^1^H COSY, HSQC, HMBC, and ROESY experiments made it possible to define all the resonances related to the steroid nucleus of **1** (Figure 2, Figure 3 and Figure S4–S7). The key ROESY cross-peaks from H-3 to Hα-1, Hα-2, and Hα-4; from Hα-4 to H-6; from H-14 to H-17; from H_3_-18 to H-8, Hβ-12; and from H_3_-19 to Hβ-1, Hβ-4, and H-8 confirmed the Δ^5^-3β-sulfoxy fragment in 9α/10β/13β/14α steroid nucleus in **1** (Figure 3 and Figure S7). The ^1^H- and ^13^C-NMR data of the side chain of **1** revealed the presense of two secondary methyls (*δ*_H_ 0.96 d, *J* = 6.7; *δ*_C_ 23.1, CH_3_-26) and (*δ*_H_ 0.96 d, *J* = 6.7; *δ*_C_ 22.9, CH_3_-27), a characteristic oxidized methylene (*δ*_H_ 4.13 dd, *J* = 9.8, 4.4, 3.83 dd, *J* = 9.8, 7.4; *δ*_C_ 71.5, CH_2_-21), and the Δ^22^ double bond (*δ*_H_ 5.24 dd, *J* = 15.4, 9.3, 5.39 dd, *J* = 15.4, 6.8; *δ*_C_ 129.5, 139.6). In the HMBC spectrum, the key correlations from H-17 to C-22; from H_2_-21 to C-17, C-20, and C-22; from H-22 to C-25; from H-23 to C-25, C-26, and C-27; from H-25 to C-22, C-23, C-26, and C-27 were observed (Figure 2 and Figure S6). Moreover, the cross-peaks from H-22 to H-17, H-25 and from H-23 to H-20, H_3_-26, and H_3_-27 were present in the ROESY spectrum (Figure 3 and Figure S7). These findings along with the HRESIMS allowed us to assume the Δ^22^-21-sulfoxy-24-norcholestane side chain in steroid **1**. The *E* configuration of the Δ^22^ double bond was determined based on the *J*_22,23_ value (15.4 Hz) in the ^1^H-NMR spectrum of **1**. We have proposed an *R* configuration of the asymmetric center C-20 based on the ROESY correlations from H_3_-18 to H-20, H_2_-21 and from H_2_-21 to Hβ-12, H-17 [17] and the similarity of the NMR spectroscopic data of **1** with those reported for related steroids from brittle stars containing the same side chains [11,14]. Therefore, the structure of compound **1** was elucidated as the (20*R*,22*E*)-24-norcholesta-5,22-diene-3β,21-diol 3,21-disulfate, disodium salt.

Steroid **2** had the molecular formula C_26_H_42_O_8_S_2_Na_2_, established from the peaks of [M − 2Na + H]^−^ ion at *m*/*z* 547.2423 and [M − 2Na]^2−^ ion at *m*/*z* 273.1174 in the (−)HRESIMS, and from the peaks of [M + Na]^+^ ion at *m*/*z* 615.2000 and [M + H]^+^ ion at *m*/*z* 593.2187 in the (+)HRESIMS. It was found that there were two sulfate groups in **2**, which followed from the HRESIMS, NMR data, and the (−)HRESIMS/MS spectrum of precursor [M − 2Na]^2−^ ion at *m/z* 273.1171, comprising peaks of fragment ions at *m/z* 449.2739 [M − 2Na − HSO_4_]^−^ and 96.9608 [HSO_4_]^−^ (Figure S8). So, the molecular weight of compound **2** was 2 amu more than that of **1**. The detailed matching of the NMR spectra of steroids **1** and **2** clearly demonstrated that both substances have the same Δ^22^-21-sulfoxy-24-norcholestane side chain and steroid **2** differs from **1** in the lack of the 5(6)-double bond in the steroid nucleus (Table 1 and Table 2). The signals of protons and carbons of angular methyls (*δ*_H_ 0.71 s, *δ*_C_ 12.6, CH_3_-18) and (*δ*_H_ 0.84 s, *δ*_C_ 13.1, CH_3_-19), and an oxidized methine (*δ*_H_ 4.25 m, *δ*_C_ 79.7, CH-3) belonging to the tetracyclic pattern of **2** were indicated in the NMR spectra (Figures S9 and S10). Analysis of the ^1^H-^1^H COSY and HSQC spectra (Figures S11 and S12) confirmed the proton spin coupling systems as shown in Figure 2. In the HMBC spectrum (Figure 2 and Figure S13), the cross-peaks from H-4 to C-3, C-5; from H-17 to C-13; from H_3_-18 to C-12, C-13, C-14, and C-17; from H_3_-19 to C-1, C-5, C-9, and C-10; and in the ROESY spectrum (Figure 3 and Figure S14), the correlations from H-3 to Hα-2, Hα-4, and H-5; from H-5 to Hα-7; from H-14 to Hα-15, H-17; from H_3_-18 to H-8, Hβ-11, Hβ-12, Hβ-16, and H-20; from H_3_-19 to Hβ-1, Hβ-4, Hβ-6, and Hβ-11; and from H-14 to Hα-15 and H-17 verified the 3β-sulfoxy-5α-cholestane nucleus in steroid **2**. On the basis of above-mentioned data, the structure of **2** was established as the (20*R*,22*E*)-24-nor-5α-cholest-22-ene-3β,21-diol 3,21-disulfate, disodium salt. In addition to highly oxidized steroid metabolites, starfish also contain low-polar Δ^7^-sterols, stanols and sterol sulfates. Among them there are compounds with shortened side chains. Previously, it was experimentally shown that the biosynthetic precursors of the natural polar steroid compounds of sea stars are nutritive cholesterol or sulfate of cholesterol [18]. It is likely that the biosynthesis of compounds **1** and **2** in starfish is carried out from 24-nor-sterols or related non-polar steroids.

Steroid **3** had the molecular formula C_28_H_44_O_8_S_2_Na_2_, determined from the peaks of [M − Na]^−^ ion at *m*/*z* 595.2392 and [M − 2Na]^2−^ ion at *m*/*z* 286.1253 in the (−)HRESIMS, and from the peak of cationized molecule [M + Na]^+^ at *m*/*z* 641.2165 in the (+)HRESIMS. It was found that there were two sulfate groups in **3**, which followed from the HRESIMS, NMR data, and the (−)HRESIMS/MS spectrum of precursor [M − 2Na]^2−^ ion at *m/z* 286.1254, containing of peaks of fragment ions at *m/z* 475.2904 [M − 2Na − HSO_4_]^−^ and 96.9610 [HSO_4_]^−^ (Figure S15). Comparison of the NMR spectroscopic data of steroids **3** and **1** indicated that the signals of protons and carbons belonging to the tetracyclic part of **3** are identical to those of **1**, that proved the cholestane nucleus with Δ^5^-3β-sulfoxy structural fragment in **3** (Figures S16 and S17). At the same time, the chemical shifts of protons and carbons of the steroid side chain of **3** were significantly different from those of **1** (Table 1 and Table 2). The signals of protons and carbons of three secondary methyls (*δ*_H_ 0.96 d, *J* = 6.2; *δ*_C_ 12.4, CH_3_-21), (*δ*_H_ 1.06 d, *J* = 6.7; *δ*_C_ 22.6, CH_3_-26), and (*δ*_H_ 1.05 d, *J* = 6.7; *δ*_C_ 21.8, CH_3_-27), an oxidized methine (*δ*_H_ 4.58 dd, *J* = 11.0, 4.0; *δ*_C_ 81.2 CH-22), and the 24(28)-double bond (*δ*_H_ 4.84 s, 4.75 s; *δ*_C_ 154.3, 110.1) of the steroid side chain were detected in the NMR spectra of **3**. The sequences of protons in the steroid side chain of **3** shown in Figure 2 were defined by the ^1^H-^1^H COSY and HSQC spectra (Figures S18 and S19). In the HMBC spectrum (Figure 2 and Figure S20), the correlations from H_3_-21 to C-17, C-20, and C-22; from H-22 to C-17, C-20, and C-24; from H-23 to C-28; and from H-25 to C-24, C-26, C-27, and C-28 confirmed the position of a sulfoxy group at C-22 and the 24(28)-double bond. Previously, the resonance of H_3_-21 of natural 20*R* steroids with a saturated cholestane side chain was reported to appear more than *δ*_H_ 0.90, whereas the same signal of synthetic 20*S* steroids with a saturated cholestane side chain to appear at 0.1 ppm shielded in the ^1^H-NMR spectra [19,20]. Moreover, it was experimentally determined that the biosynthetic precursors of the natural polar steroid compounds of sea stars are nutritive cholesterol or sulfate of cholesterol, having a 20*R*-configuration [18]. The deshielded signal of H_3_-21 at *δ*_H_ 0.96 (*δ*_H_ 0.92 for natural and synthetic 20*S*,22*R*-22-hydroxy steroids [21,22] and *δ*_H_ 0.79 for synthetic 20*R*,22*R*-22-hydroxy steroid [23]) and the presence of the ROESY cross-peaks from H_3_-18 to H-20, H_3_-21; from H_3_-21 to Hβ-12, H-17; and from H-22 to H_2_-16 [17] suggested the 20*S*,22*R* configurations in **3** (Figure 3 and Figure S21). Earlier similar NOEs cross-peaks were observed for 3-keto-22-epi-28-nor-cathasterone with a (20*S*,22*R*)-22-hydroxycholestane side chain from the brown alga *Cystoseira myrica* [21]. This also confirmed our assumption. Consequently, the structure of **3** was established as (20*S*,22*R*)-24-methylcholesta-5,24-diene-3β,22-diol 3,22-disulfate, disodium salt. To the best of our knowledge, the structurally related 24-methylcholesta-5,24-diene-3β,22*R*-diol has previously been isolated from leaves and fruits of *Trichilia pallida* Swartz [24].

Steroid **4** had the molecular formula C_28_H_46_O_9_S_2_Na_2_, established from the peaks of [M − Na]^−^ ion at *m*/*z* 613.2477 and [M − 2Na]^2−^ ion at *m*/*z* 295.1292 in the (−)HRESIMS, and from the peak of cationized molecule [M + Na]^+^ at *m*/*z* 659.2271 in the (+)HRESIMS. The HRESIMS, NMR data and the (−)HRESIMS/MS spectrum of precursor [M − 2Na]^2−^ ion at *m/z* 295.1280, comprising peaks of fragment ions at *m/z* 493.2973 [M − 2Na − HSO_4_]^−^ and 96.9609 [HSO_4_]^−^, indicated the existence of two sulfate groups in compound **4** (Figure S22). Based on the NMR spectra of **3** and **4**, both steroids contain the same Δ^24(28)^-22-sulfoxy-24-methylcholestane side chain, but the chemical shifts of protons and carbons of the tetracyclic pattern of **4** differed from those of **3** (Table 1 and Table 2; Figures S23 and S24). Analysis of HRESIMS and NMR data of **3** and **4** showed the absence of a 5(6)-double bond and the appearance of an additional hydroxyl group in the tetracyclic core of **4** in comparison with **3**.

In the NMR spectra, the resonances of protons and carbons of two angular methyls (*δ*_H_ 0.64 s, *δ*_C_ 12.3, CH_3_-18) and (*δ*_H_ 0.98 s; *δ*_C_ 14.6, CH_3_-19), two oxidized methines (*δ*_H_ 4.07 br d, *J* = 2.5; *δ*_C_ 70.0, CH-2) and (*δ*_H_ 4.39 br d, *J* = 2.5; *δ*_C_ 78.5, CH-3) associated with steroid nucleus of **4** were present. The coupling constants and proton chemical shifts of H-2, H-3, H_3_-18, and H_3_-19 and the carbon signals C-1–C-19 closely resembled those reported for (20*R*)-5α-cholestane-2β,3α,21-triol 3,21-disulfate from the starfish *P. pulvillus* [16] and (20*R*)-5α-cholest-24-ene-2β,3α,21-triol 3,21-disulfate from the ophiuroid *Astrotoma agassizii* [13]. The equatorial orientation of protons at the oxygenated carbons C-2 and C-3 followed from the small *J*_2,3_ (2.5 Hz) value in the ^1^H-NMR spectrum of **4**. In addition, the characteristic signals of C-1 (*δ*_C_ 41.0) and C-4 (*δ*_C_ 30.3) in the ^13^C-NMR spectrum of **4** differed from the corresponding values of C-1 (*δ*_C_ 38.9) and C-4 (*δ*_C_ 32.5) reported for (20*R*)-5α-cholestane-2β,3α,21-triol 2,21-disulfate from *A. agassizii* [13]. It also confirmed the availability of a sulfate group at position C-3 in **4**. Based on these data, we assumed that steroid **4** has a 2β-hydroxy-3α-sulfoxy-5α-cholestane nucleus. Examination of 2D NMR spectra revealed a total chemical structure of compound **4** (Figure 2, Figure 3 and Figure S25–S28). The downfield resonance of H_3_-21 at *δ*_H_ 0.95 and the ROESY correlations from H_3_-18 to H-20, H_3_-21; from H_3_-21 to Hβ-12, H-17; and from H-22 to H_2_-16 supported the 20*S*,22*R* configurations in **4** by analogy with steroid **3**. Accordingly, the structure of **4** was proposed as (20*S*,22*R*)-24-methyl-5α-cholest-24-ene-2β,3α,22-triol 3,22-disulfate, disodium salt. The Δ^24(28)^-22-sulfoxy-24-methylcholestane side chain of **3** and **4** is first discovered in the steroid substances from sea stars and brittle stars.

In addition to steroid disulfates **1**–**4**, the non-steroid metabolite tryptamine (**5**) was found in the sea star *P. marsippus*. The peak of protonated molecule [M + H]^+^ at *m*/*z* 161.1073 in the (+)HRESIMS (Figure S29) exhibited the molecular formula of **5** was C_10_H_12_N_2_. The ^13^C-NMR and DEPT spectra indicated the availability of 10 carbons in **5**, including 2 methylenes, 5 methines, and 3 carbons unbound to the protons (Figures S31 and S32). The ^1^H-^1^H COSY and HSQC spectra showed the aromatic proton structural fragment from C-4 to C-7 and bonding of protons from C-2 to C-β (Figures S33 and S34). The main HMBC cross-peaks from H-2 to C-3, C-9; from H-4 to C-3, C-6, and C-8; from H-6 to C-4, C-8; from H-7 to C-5, C-9; from H-α to C-3, C-β; and from H-β to C-2, C-3, C-9, and C-α and the main ROESY correlations from H-4 to H-α, and H-β; and from H-2 to H-α, H-β confirmed the indolamine sceleton in **2** (Figures S35 and S36). As a result, the analysis of the NMR spectra and the HRESIMS led to the identification of compound **2** as tryptamine. The previously known tryptamine, a monoamine alkaloid, was found in animals, humans, and plants, although it was first discovered in starfish. Alkaloids have not often been found in starfish. For instance, tyramine and salsolinol as cations in salts of sulfated steroids and 1-methyl-1,2,3,4-tetrahydro-β-carboline-3-carboxylic acid (MTSA) were isolated from the sea star *Lethasterias nanimensis chelifera* [25]. In addition, imbricatin [26], ovothiol A [27], and a number of guanidine metabolites [28] were found in some species of starfish.

### 2.2. The Effect of Compounds **1**–**4** on Cell Viability of Human Breast Cancer Cells

**Figure 4:**
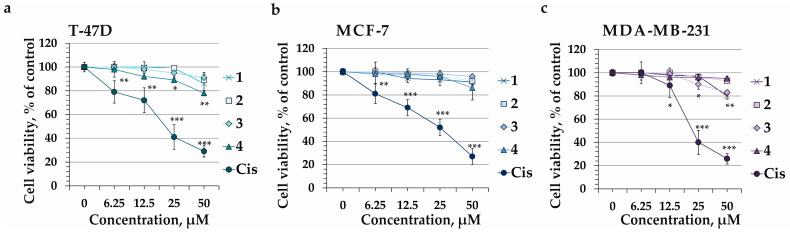
The cytotoxic activity of compounds **1**–**4** against human breast cancer cells. (**a**) T-47D, (**b**) MCF-7, and (**c**) MDA-MB-231 cells were incubated with **1**–**4** (6.25, 12.5, 25, and 50 µM) or cisplatin (Cis) (6.25, 12.5, 25, and 50 µM) for 72 h. MTS assay was used to evaluate cytotoxicity of compounds. The results are presented as the mean ± SD for triplicate experiments. The asterisk (*) indicates a significant decrease in cell viability of cancer cells treated with different concentrations of investigated compounds at a time point 72 h as compared to control (* *p* < 0.05, ** *p* < 0.01, *** *p* < 0.001).

The effect of compounds **1**–**4** on cell viability of human breast cancer cells T-47D, MCF-7, and MDA-MB-231 was determined at 24, 48, and 72 h of treatment. MTS assay revealed that compounds **1**–**4** possessed less cytotoxic effect on the cell viability of tested cancer cells even after 72 h of cell incubation (Figure 4). Thus, compounds **1**, **2**, **3**, and **4** at a concentration of 50 µM inhibited the cell viability of T-47D cells by 14%, 11%, 9%, and 22%, respectively (Figure 4a); MCF-7 cell line—by 10%, 8%, 3%, and 18%, respectively (Figure 4b); and MDA-MB-231 cells—by 6%, 7%, 17%, and 5%, respective (Figure 4c). Herein, the cisplatin, which is a well-known chemotherapeutic drug for treatment of numerous types of human cancers, was used as a positive control. It was shown that cisplatin greatly suppressed the cell growth of T-47D (IC_50_ of 17 µM), MCF-7 (IC_50_ of 22 µM), and MDA-MB-231 (IC_50_ of 18 µM) after 72 h of cell treatment (Figure 4).

The lack of high cytotoxicity of compounds **1**–**4** confirmed their safety, so they were used at non-toxic concentrations of 12.5, 25, and 50 µM for the following assays.

### 2.3. The Effect of Compounds **1**–**4** on Colony Formation and Growth of Human Breast Cancer Cells

**Figure 5:**
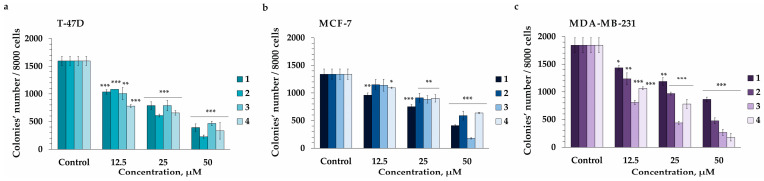
The colony-inhibiting activity of compounds **1**–**4** in human breast cancer cells. (**a**) T-47D, (**b**) MCF-7, and (**c**) MDA-MB-231 cells were treated by **1**–**4** (12.5, 25, and 50 µM) in soft agar. Microscope (at a total magnification of 40×) using the ImageJ software version 1.50i bundled with 64 bit Java 1.6.0_24 (“NIH”, Bethesda, MD, USA) were used to count the number of colonies. Results are presented as the mean ± standard deviation (SD). A one-way ANOVA and Tukey’s HSD test for multiple comparisons indicated the statistical significance (* *p* < 0.05, ** *p* < 0.01, *** *p* < 0.001).

The sustained proliferation of cancer cells is an important hallmark of carcinogenesis [29]. Malignant cancer cells are capable of proliferating and growing without attachment to a substrate and formed colonies. The tumorigenic potential of cancer cell and anticancer efficacy of potential drugs can be assessed using soft agar colony formation assay which uniquely detect anchorage-independent growth of malignant cells [30]. In this work the soft agar assay was applied to assess the colony-inhibiting activity of compounds **1**–**4**. All the investigated compounds were found to significantly decrease colonies number dose-dependently (Figure 5). Compound **1** at concentrations of 12.5, 25, and 50 µM inhibited colony formation in T-47D cells by 35%, 51%, and 76%, respectively; MCF-7—by 28%, 44%, and 60%, respectively; and MDA-MB-231—by 22%, 36%, and 53%, respectively (Figure 5). Compound **2** at concentrations of 12.5, 25, and 50 µM inhibited colony formation of T-47D cells by 32%, 62%, and 86%, respectively; MCF-7—by 24%, 32%, and 56%, respectively; and MDA-MB-231—by 33%, 47%, and 74%, respectively (Figure 5). Compound **3** at concentrations of 12.5, 25, and 50 µM inhibited colony formation of T-47D cells by 37%, 51%, and 71%, respectively; MCF-7—by 15%, 34%, and 87%, respectively; and MDA-MB-231—by 56%, 76%, and 86%, respectively (Figure 5). Compound **4** at concentrations of 12.5, 25, and 50 µM inhibited colony formation of T-47D cells by 51%, 59%, and 79%, respectively; MCF-7—by 18%, 33%, and 52%, respectively; and MDA-MB-231—by 42%, 58%, and 90%, respectively (Figure 5). Thus, it was found that compounds **1** and **2** possessed significant colony-inhibiting activity against T-47D cells, while compounds **3** and **4** were more effective against MDA-MB-231 cells (Figure 5).

## 3. Materials and Methods

### 3.1. General Procedures

Optical rotations, Perkin-Elmer 343 polarimeter (PerkinElmer, Waltham, MA, USA). NMR spectra, Bruker Avance III 700 spectrometer (Bruker BioSpin, Bremen, Germany) at 700.13 MHz (^1^H)/176.04 MHz (^13^C), internal standard CD_3_OD at *δ*_H_ 3.30/*δ*_C_ 49.0. HRESIMS spectra, Bruker Impact II Q-TOF mass spectrometer (Bruker, Bremen, Germany); sample concentration in MeOH 0.001 mg/mL. HPLC, Agilent 1100 Series chromatograph (Agilent Technologies, Santa Clara, CA USA) with a differential refractometer; columns Discovery C18 (5 µm, 10.0 × 250 mm, Supelco, Bellefonte, PA, USA) and YMC-Pack Pro C18 (5 µm, 10.0 × 250 mm and 4.6 × 250 mm, YMC Co., Ltd., Kyoto, Japan). LPLC, column sorbents Polychrom 1 (powdered Teflon, 0.25–0.50 mm, Biolar, Olaine, Latvia), Si gel KSK (50–160 µm, Sorbpolimer, Krasnodar, Russia), and Florisil (60–100 µm, Sigma-Aldrich, Co., St. Louis, MO, USA).

### 3.2. Animal Material

Specimens of *Pteraster marsippus* Fisher, 1910 (order Velatida, family Pterasteridae) were collected near Urup Island (Sea of Okhotsk) at a depth of 84–88 m using a small trawl (research vessel *Akademik Oparin*, 51st scientific cruise, May 2017). Taxonomical identification of species was determined by Mr. Boris B. Grebnev. A voucher specimen was deposited in G.B. Elyakov PIBOC FEB RAS, Vladivostok, Russia.

### 3.3. Extraction and Isolation

The concentrated ethanol extract of the sliced specimens (2.1 kg) of the immediately frozen after fishing sea star *P. marsippus* was chromatographed on a column with Polychrome 1. The total fraction of steroid compounds were eluted with 50% aq. EtOH and separated by sequential chromatography on Si gel and Florisil columns using CHCl_3_/EtOH/H_2_O (stepwise gradient) eluent systems to yield 11 fractions (fr.1–fr.11) as previously reported in [15]. Fr. 8 (77.5 mg) was separated by HPLC on a Discovery C18 column (MeOH/H_2_O/1M NH_4_OAc, 70:29:1, *v*/*v*/*v*, flow rate: 2.0 mL/min) and purified repeatedly under the same conditions yielded **1** (2.3 mg, *t*_R_ 10.7 min), **2** (1.7 mg, *t*_R_ 15.5 min), and **5** (7.4 mg, *t*_R_ 8.0 min). HPLC of fr. 9 (87.0 mg) on a Discovery C18 column (60% aq. MeOH, flow rate: 2.0 mL/min) gave subfractions 9-1 and 9-2. HPLC of subfraction 9-1 on a semi-preparative YMC-Pack Pro C18 column (75% aq. MeOH, flow rate: 1.8 mL/min) afforded **3** (1.4 mg, *t*_R_ 11.7 min). HPLC of subfraction 9-2 on an analytical YMC-Pack Pro C18 column (60% aq. MeOH, flow rate: 0.5 mL/min) yielded **4** (1.2 mg, *t*_R_ 22.0 min).

### 3.4. Compound Characterization Data

Disodium salt of (20*R*,22*E*)-24-norcholesta-5,22-diene-3β,21-diol 3,21-disulfate (**1**): colorless powder; [α]_D_^25^: –9.7 (*c* 0.23, MeOH); (−)HRESIMS *m/z* 567.2073 [M − Na]^−^ (calcd for C_26_H_40_O_8_S_2_Na, 567.2068); (−)HRESIMS *m/z* 272.1090 [M − 2Na]^2−^ (calcd for C_26_H_40_O_8_S_2_, 272.1088); (+)HRESIMS *m/z* 613.1847 [M + Na]^+^ (calcd for C_26_H_40_O_8_S_2_Na_3_, 613.1852); (−)HRESIMS/MS of the [M − 2Na]^2−^ ion at *m/z* 272.1076: 447.2561 [M − 2Na − HSO_4_]^−^, 96.9611 [HSO_4_]^−^; ^1^H-NMR data, see Table 1; ^13^C-NMR data, see Table 2.

Disodium salt of (20*R*,22*E*)-24-nor-5α-cholest-22-ene-3β,21-diol 3,21-disulfate (**2**): colorless powder; [α]_D_^25^: –1.4 (*c* 0.14, MeOH); (−)HRESIMS *m/z* 547.2423 [M − 2Na + H]^−^ (calcd for C_26_H_43_O_8_S_2_, 547.2405); (−)HRESIMS *m/z* 273.1174 [M − 2Na]^2−^ (calcd for C_26_H_42_O_8_S_2_, 273.1166); (+)HRESIMS *m/z* 615.2000 [M + Na]^+^ (calcd for C_26_H_42_O_8_S_2_Na_3_, 615.2009); (+)HRESIMS *m/z* 593.2187 [M + H]^+^ (calcd for C_26_H_43_O_8_S_2_Na_2_, 593.2165); (−)HRESIMS/MS of the [M − 2Na]^2−^ ion at *m/z* 273.1171: 449.2739 [M − 2Na − HSO_4_]^−^, 96.9608 [HSO_4_]^−^; ^1^H-NMR data, see Table 1; ^13^C-NMR data, see Table 2.

Disodium salt of (20*S*,22*R*)-24-methylcholesta-5,24-diene-3β,22-diol 3,22-disulfate (**3**): colorless powder; [α]_D_^25^: –28.8 (*c* 0.04, MeOH); (−)HRESIMS *m/z* 595.2392 [M − Na]^−^ (calcd for C_28_H_44_O_8_S_2_Na, 595.2381); (−)HRESIMS *m/z* 286.1253 [M − 2Na]^2−^ (calcd for C_28_H_44_O_8_S_2_, 286.1244); (+)HRESIMS *m/z* 641.2165 [M + Na]^+^ (calcd for C_28_H_44_O_8_S_2_Na_3_, 641.2165); (−)HRESIMS/MS of the [M − 2Na]^2−^ ion at *m/z* 286.1254: 475.2904 [M − 2Na − HSO_4_]^−^, 96.9610 [HSO_4_]^−^; ^1^H-NMR data, see Table 1; ^13^C-NMR data, see Table 2.

Disodium salt of (20*S*,22*R*)-24-methyl-5α-cholest-24-ene-2β,3α,22-triol 3,22-disulfate (**4**): colorless powder; [α]_D_^25^: +1.7 (*c* 0.12, MeOH); (−)HRESIMS *m/z* 613.2477 [M − Na]^−^ (calcd for C_28_H_46_O_9_S_2_Na, 613.2486); (−)HRESIMS *m/z* 295.1292 [M − 2Na]^2−^ (calcd for C_28_H_46_O_9_S_2_, 295.1297); (+)HRESIMS *m/z* 659.2271 [M + Na]^+^ (calcd for C_28_H_46_O_9_S_2_Na_3_, 659.2271); (−)HRESIMS/MS of the [M − 2Na]^2−^ ion at *m/z* 295.1280: 493.2973 [M − 2Na − HSO_4_]^−^, 96.9609 [HSO_4_]^−^; ^1^H-NMR data, see Table 1; ^13^C-NMR data, see Table 2.

Tryptamine (**5**): colorless powder; [α]_D_^20^ 0 (*c* 0.1, MeOH); (+)HRESIMS *m/z* 161.1073 [M + H]^+^ (calcd for C_10_H_13_N_2_, 161.1073); ^1^H-NMR (CD_3_OD, 500.13 MHz): *δ*_H_ 3.10 (2H, t, *J* = 7.3 Hz, H_2_-β), 3.21 (2H, t, *J* = 7.3 Hz, H_2_-α), 7.03 (1H, t, *J* = 7.5 Hz, H-5), 7.12 (1H, t, *J* = 7.5 Hz, H-6), 7.15 (1H, s, H-2), 7.36 (1H, d, *J* = 8.0 Hz, H-7), 7.56 (1H, d, *J* = 8.0 Hz, H-4); ^13^C-NMR (CD_3_OD, 125.76 MHz): *δ*_C_ 124.2 (C-2), 110.6 (C-3), 118.9 (C-4), 120.0 (C-5), 122.7 (C-6), 112.5 (C-7), 138.4 (C-8), 128.2 (C-9), 41.5 (C-α), 25.1 (C-β).

### 3.5. Reagents

Roswell Park Memorial Institute Medium (RPMI 1640), Dulbecco’s Modified Eagle Medium (DMEM), phosphate-buffered saline (PBS), L-glutamine, penicillin–streptomycin solution, trypsin, fetal bovine serum (FBS), sodium hydrocarbonate (NaHCO_3_) and agar were purchased from “Biolot” (Russia).

MTS reagent—3-[4,5-dimethylthiazol-2-yl]-2,5-diphenyltetrazolium bromide was purchased from “Promega” (Madison, WI, USA). Cisplatin was purchased from VeroPharm (Moscow, Russia).

### 3.6. Cell Lines

Human breast cancer T-47D (ATCC^®^ no. HTB-133™), MCF-7 (ATCC^®^ no. HTB-22™), and MDA-MB-231 (ATCC^®^ no. HTB-26™) cell lines were purchased from ATCC (Manassas, VA, USA).

### 3.7. Cell Culture Assay

Human breast cancer T-47D cells were grown in monolayer in RPMI-1640 medium, while MCF-7 and MDA-MB-231 cells were cultured in DMEM medium according to the manufacturer’s instructions. Medium were supplemented with 10% heat-inactivated fetal bovine serum (FBS) and 1% penicillin–streptomycin solution. The cells were cultured at 37 °C in humidified atmosphere containing 5% CO_2_. Cells were routinely checked for contamination with mycoplasma.

### 3.8. Cytotoxicity Assay

The effect of compounds **1**–**4** on cell viability was determined through mitochondrial-dependent reduction of formazan using MTS reagent. Cells were seeded at a density of 1.0 × 10^4^ cells/200 µL of complete medium into 96-well plates and cultured for 24 h. Attached cells were treated with cisplatin at concentrations of 6.25, 12.5, 25, and 50 µM or compounds **1**–**4** at concentrations of 6.25, 12.5, 25, and 50 µM, while the control was treated with the complete RPMI-1640 or DMEM medium only. Cells were cultured for additional 24, 48, and 72 h at 37 °C in 5% CO_2_ incubator. Then, cells were incubated with MTS-reagent (20 µL) for 3 h at 37 °C in 5% CO_2_. Absorbance was measured at 490/630 nm by microplate reader (Power Wave XS, city Winooski, VT, USA). All tested samples were carried out in triplicates. Data on relative cell viability were expressed in terms of percentage of the non-treated control cells.

### 3.9. Colony-Formation Assay (Soft Agar Assay)

Cells (2.4 × 10^4^/mL) were applied onto Agar Mix (0.3% BME agar, 10% FBS, 2 mM L-glutamine, and 25 µg/mL gentamicin) containing **1**–**4** (12.5, 25, and 50 µM). The colony formation of human breast cancer cells was detected after 14 days of incubation at 37 °C in a 5% CO_2_ incubator. Motic microscope AE 20 and ImageJ software bundled with 64 bit Java 1.8.0_112 (NIH, Bethesda, MD, USA) were used to count the number of colonies of cancer cells tested.

### 3.10. Statistical Analysis

All of the assays were performed in at least three independent experiments. Results are expressed as the mean ± standard deviation (SD). Statistical procedures were performed using one-way ANOVA and Tukey’s HSD tests with * *p* < 0.05, ** *p* < 0.01, and *** *p* < 0.001.

## 4. Conclusions

Two new steroid 3β,21-disulfates, two new steroid 3β,22- and 3α,22-disulfates, and a known monoamine alkaloid tryptamine were obtained from the slime sea star *Pteraster marsippus* collected in the Sea of Okhotsk. Two compounds have a shortened Δ^22^-21-sulfoxy-24-norcholestane side chain. Two other steroids include a Δ^24(28)^-22-sulfoxy-24-methylcholestane side chain, which was first discovered in polar steroids of starfish and ophiuroids. Thus, taking into account the previous publication, nine steroid disulfates of the ophiuroid type were isolated from *P. marsippus*, of which eight compounds turned out to be new. This brings the number of new steroid disulfates from starfish of the Pterasteridae family to twenty-four. At the same time, the usual steroid metabolites of starfish, such as polyhydroxysteroids and asterosaponins, were not found in *P. marsippus*, as in other species of this family previously studied. The structural similarity of steroid disulfates from the representatives of the Pterasteridae family and ophiuroids appears to indicate the phylogenetic closeness of the classes Asteroidea and Ophiuroidea as opposed to the classes Holothurioidea, Echinoidea, and Ophiuroidea of echinoderms. The performed soft agar assays demonstrated that new steroid disulfates significantly inhibited the colony formation and growth of human breast cancer cells at non-toxic concentrations.

In addition, a known monoamine alkaloid tryptamine was obtained from *P. marsippus* in quantities comparable to steroid substances. Tryptamine was discovered in the starfish for the first time. Although some alkaloids were previously isolated from several species of the starfish both in the free form and in the form of salts with sulfated steroids as cations, this finding probably indicates the importance of tryptamine in the physiology of *P. marsippus.*

## Figures and Tables

**Table 1:** ^1^H-NMR (CD_3_OD, 700.13 MHz) data of compounds **1**−**4** (*δ* in ppm, *J* in Hz).

Position	1	2	3	4
1β α	1.89 m 1.10 m	1.75 dt (13.4, 3.5) 1.01 m	1.89 m 1.10 m	1.75 m 1.36 m
2α β	2.04 m 1.62 m	2.00 m 1.52 m	2.05 m 1.61 m	4.07 br d (2.5)
3	4.13 m (1/2 Δ*W* = 19.8 Hz)	4.25 m	4.13 m	4.39 br d (2.5)
4α β	2.53 ddd (13.4, 4.8, 2.2) 2.33 m	1.80 m 1.40 m	2.53 ddd (13.3, 4.9, 2.2) 2.34 m	1.81 m 1.59 m
5	–	1.14 m	–	1.58 m
6	5.38 m	1.28 m	5.38 m	1.24 m
7β α	1.96 m 1.54 m	1.67 m 0.90 m	1.96 m 1.54 m	1.67 m 0.93 m
8	1.47 m	1.38 m	1.46 m	1.38 m
9	0.95 m	0.67 m	0.95 m	0.70 m
10	–	–	–	–
11	1.52 m 1.02 m	1.53 m 1.32 m	1.53 m 1.04 m	1.52 m 1.31 m
12β α	1.99 m 1.20 m	1.95 dt (12.6, 3.0) 1.17 m	2.01 m 1.24 m	1.96 m 1.18 m
13	–	–	–	–
14	1.05 m	1.04 m	1.08 m	1.09 m
15α β	1.60 m 1.08 m	1.58 m 1.07 m	1.62 m 1.09 m	1.59 m 1.07 m
16α β	1.66 m 1.37 m	1.64 m 1.32 m	2.25 m 1.15 m	2.22 m 1.14 m
17	1.44 m	1.44 m	1.64 m	1.64 m
18	0.75 s	0.71 s	0.67 s	0.64 s
19	1.03 s	0.84 s	1.02 s	0.98 s
20	2.31 m	2.30 m	1.63 m	1.64 m
21	4.13 dd (9.8, 4.4) 3.83 dd (9.8, 7.4)	4.13 dd (9.4, 4.4) 3.81 dd (9.4, 7.6)	0.96 d (6.2)	0.95 d (6.0)
22	5.24 dd (15.4, 9.3)	5.24 dd (15.3, 9.8)	4.58 dd (11.0, 4.0)	4.57 dd (11.0, 4.2)
23	5.39 dd (15.4, 6.8)	5.38 dd (15.3, 6.8)	2.85 dd (13.7, 4.0) 2.35 dd (13.7, 11.0)	2.85 dd (13.7, 3.8) 2.35 dd (13.7, 11.0)
24	–	–	–	–
25	2.22 m	2.22 m	2.25 m	2.26 m
26	0.96 d (6.7)	0.96 d (6.9)	1.06 d (6.7)	1.05 d (6.8)
27	0.96 d (6.7)	0.96 d (6.9)	1.05 d (6.7)	1.04 d (6.8)
28			4.84 s 4.75 s	4.84 s 4.74 s

**Table 2:** ^13^C-NMR (CD_3_OD, 176.04 MHz) data of compounds **1**−**4** (*δ* in ppm, *J* in Hz).

Position	1	2	3	4
1	38.5	38.2	38.3	41.0
2	30.0	29.8	29.9	70.0
3	79.9	79.7	79.8	78.5
4	40.4	36.4	40.3	30.3
5	141.6	46.3	141.5	40.6
6	123.2	29.9	123.2	29.3
7	33.0	33.2	32.9	33.2
8	33.2	36.9	33.2	36.4
9	51.9	55.8	51.5	56.5
10	37.9	36.5	37.6	36.4
11	22.1	22.2	22.1	22.0
12	40.2	40.4	40.9	41.3
13	43.3	43.6	43.3	43.7
14	58.1	57.8	57.9	57.7
15	25.1	25.0	25.3	25.3
16	28.6	28.6	29.1	29.1
17	51.9	52.1	53.1	53.3
18	12.9	12.6	12.0	12.3
19	19.7	13.1	19.6	14.6
20	46.1	46.1	38.5	38.6
21	71.5	71.5	12.4	12.5
22	129.5	129.6	81.2	81.3
23	139.6	139.5	38.2	38.3
24	–	–	154.3	154.3
25	32.4	32.4	34.4	34.4
26	23.1	23.1	22.6	22.7
27	22.9	22.9	21.8	21.9
28			110.1	110.2

## Data Availability

The data presented in this study are available on request from the corresponding authors.

## Supplementary Materials

The following supporting information can be downloaded at: https://www.mdpi.com/article/10.3390/md22010043/s1, HRESIMS (Figures S1, S8, S15, S22 and S29), ^1^H-NMR (Figures S2, S9, S16, S23 and S30), ^13^C-NMR (Figures S3, S10, S17, S24 and S31), ^1^H-^1^H COSY (Figures S4, S11, S18, S25 and S33), HSQC (Figures S5, S12, S19, S26 and S34), HMBC (Figures S6, S13, S20, S27 and S35), and ROESY (Figures S7, S14, S21, S28 and S36) spectra of compounds **1**, **2**, **3, 4**, and **5**, accordingly. DEPT (Figure S32) spectrum of compound **5**.
