# Supplementary material for: Rare Ophiuroid-Type Steroid 3β,21-, 3β,22-, and 3α,22-Disulfates from the Slime Sea Star Pteraster marsippus and Their Colony-Inhibiting Effects against Human Breast Cancer Cells
Source: Mar Drugs. 2024 Jan 12;22(1):43. doi: 10.3390/md22010043 (PMC10820953; doi:10.3390/md22010043)

Supplementary Materials

Rare Ophiuroid Type Steroid 3β,21-, 3β,22-, and 3α,22-Disulfates from the Slime Sea Star *Pteraster marsippus* and Their Colony-Inhibiting Effects Against Human Breast Cancer Cells

Alla A. Kicha[1*], Timofey V. Malyarenko[1,2], Alexandra S. Kuzmich[1], Olesya S. Malyarenko[1], Anatoly I. Kalinovsky[1], Roman S. Popov[1], Dmitriy K. Tolkanov[1] and Natalia V. Ivanchina[1*]

[1] G.B. Elyakov Pacific Institute of Bioorganic Chemistry, Far Eastern Branch, Russian Academy of Sciences, Pr. 100-let Vladivostoku 159, 690022 Vladivostok, Russia; malyarenko-tv@mail.ru (T.V.M.); assavina@mail.ru (A.S.K.); malyarenko.os@gmail.com (O.S.M.); kaaniw@piboc.dvo.ru (A.I.K.); prs_90@mail.ru (R.S.P.); tolkanov.dk@gmail.com (D.K.T.);

[2] Department of Bioorganic Chemistry and Biotechnology, School of Natural Sciences, Far Eastern Federal University, Russky Island, Ajax Bay, 10, 690922, Vladivostok, Russia

* Correspondence: kicha@piboc.dvo.ru (A.A.K.); ivanchina@piboc.dvo.ru (N.V.I.). Tel.: +7-423-2312-360; Fax: +7-423-2314-050

List

	Page
**Figure S1.** HRESIMS and HRESIMS/MS spectra of compound **1**	4
**Figure S2.** ^{1}H NMR (700.13 MHz, CD_3OD) spectrum of compound **1**	5
**Figure S3.** ^{13}C NMR (176.04 MHz, CD_3OD) spectrum of compound **1**	6
**Figure S4.** ^{1}H-^{1}H COSY (700.13 MHz, CD_3OD) spectrum of compound **1**	7
**Figure S5.** HSQC (700.13 MHz, CD_3OD) spectrum of compound **1**	8
**Figure S6.** HMBC (700.13 MHz, CD_3OD) spectrum of compound **1**	9
**Figure S7.** ROESY (700.13 MHz, CD_3OD) spectrum of compound **1**	10
**Figure S8.** HRESIMS and HRESIMS/MS spectra of compound **2**	11
**Figure S9.** ^{1}H NMR (700.13 MHz, CD_3OD) spectrum of compound **2**	12
**Figure S10.** ^{13}C NMR (176.04 MHz, CD_3OD) spectrum of compound **2**	13
**Figure S11.** ^{1}H-^{1}H COSY (700.13 MHz, CD_3OD) spectrum of compound **2**	14
**Figure S12.** HSQC (700.13 MHz, CD_3OD) spectrum of compound **2**	15
**Figure S13.** HMBC (700.13 MHz, CD_3OD) spectrum of compound **2**	16
**Figure S14.** ROESY (700.13 MHz, CD_3OD) spectrum of compound **2**	17
**Figure S15.** HRESIMS and HRESIMS/MS spectra of compound **3**	18
**Figure S16.** ^{1}H NMR (700.13 MHz, CD_3OD) spectrum of compound **3**	19
**Figure S17.** ^{13}C NMR (176.04 MHz, CD_3OD) spectrum of compound **3**	20
**Figure S18.** ^{1}H-^{1}H COSY (700.13 MHz, CD_3OD) spectrum of compound **3**	21
**Figure S19.** HSQC (700.13 MHz, CD_3OD) spectrum of compound **3**	22
**Figure S20.** HMBC (700.13 MHz, CD_3OD) spectrum of compound **3**	23
**Figure S21.** ROESY (700.13 MHz, CD_3OD) spectrum of compound **3**	24
**Figure S22.** HRESIMS and HRESIMS/MS spectra of compound **4**	25
**Figure S23.** ^{1}H NMR (500.13 MHz, CD_3OD) spectrum of compound **4**	26
**Figure S24.** ^{13}C NMR (176.04 MHz, CD_3OD) spectrum of compound **4**	27
**Figure S25.** ^{1}H-^{1}H COSY (700.13 MHz, CD_3OD) spectrum of compound **4**	28
**Figure S26.** HSQC (700.13 MHz, CD_3OD) spectrum of compound **4**	29
**Figure S27.** HMBC (700.13 MHz, CD_3OD) spectrum of compound **4**	30
**Figure S28.** ROESY (700.13 MHz, CD_3OD) spectrum of compound **4**	31
**Figure S29.** HRESIMS spectrum of compound **5**	32
**Figure S30.** ^{1}H NMR (700.13 MHz, CD_3OD) spectrum of compound **5**	33
**Figure S31.** ^{13}C NMR (176.04 MHz, CD_3OD) spectrum of compound **5**	34
**Figure S32.** DEPT (176.04 MHz, CD_3OD) spectrum of compound **5**	35

Figure S33. COSY (700.13 MHz, CD_3OD) spectrum of compound **5** 36
Figure S34. HSQC (700.13 MHz, CD_3OD) spectrum of compound **5** 37
Figure S35. HMBC (700.13 MHz, CD_3OD) spectrum of compound **5** 38
Figure S36. ROESY (700.13 MHz, CD_3OD) spectrum of compound **5** 39

Figure S1. HRESIMS and HRESIMS/MS spectra of compound **1**.

(–)HRESIMS: $[M – Na]^-$ and $[M – 2Na]^{2–}$ ions

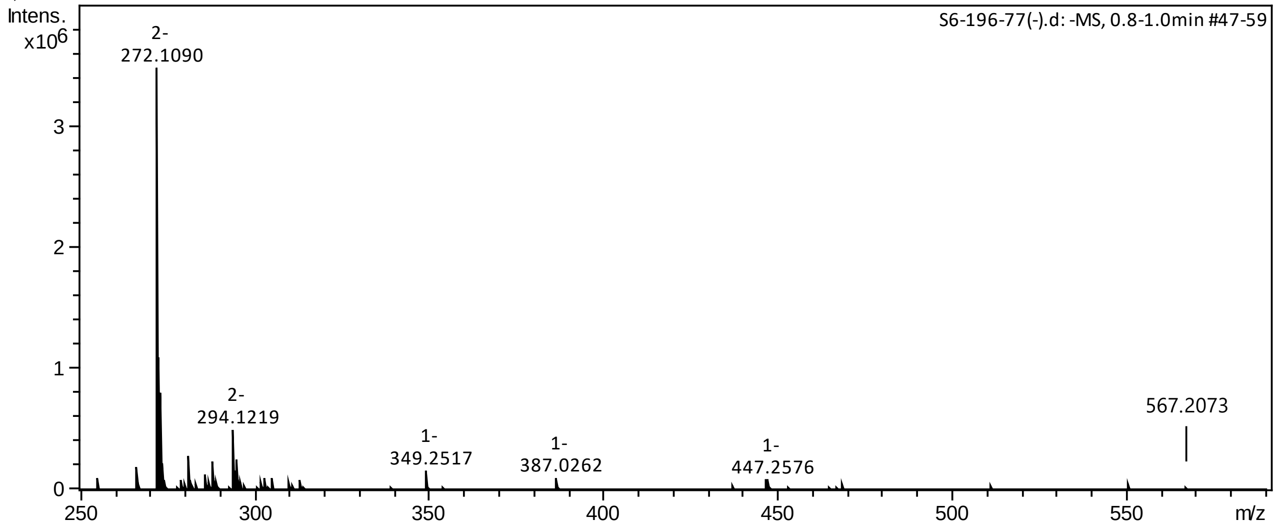

(+)HRESIMS: $[M + Na]^+$ ion

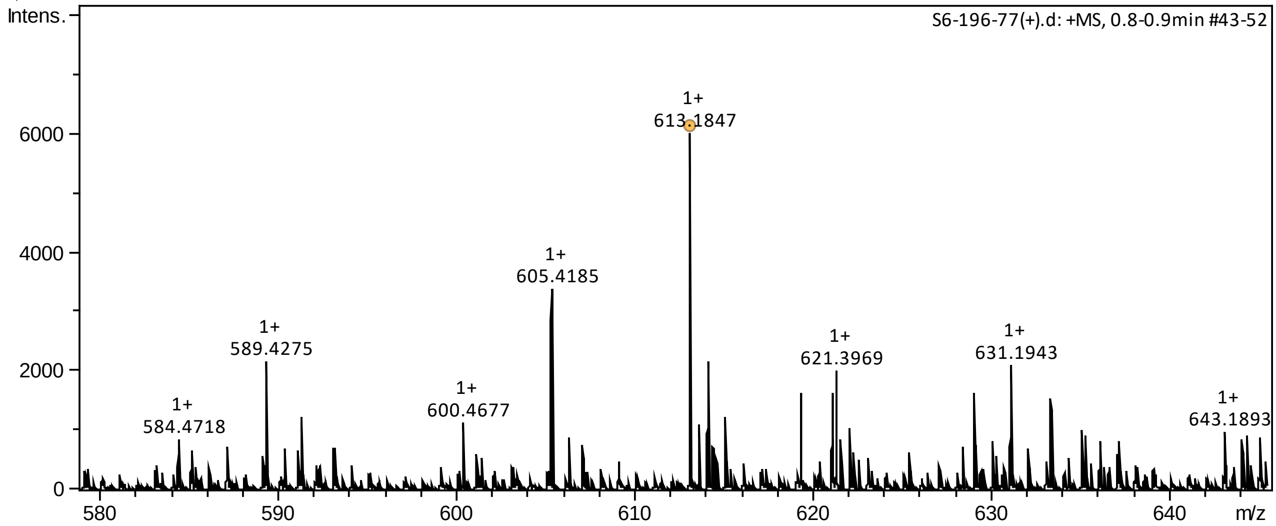

(–)HRESIMS/MS of the $[M – 2Na]^{2–}$ ion at *m/z* 272.1076

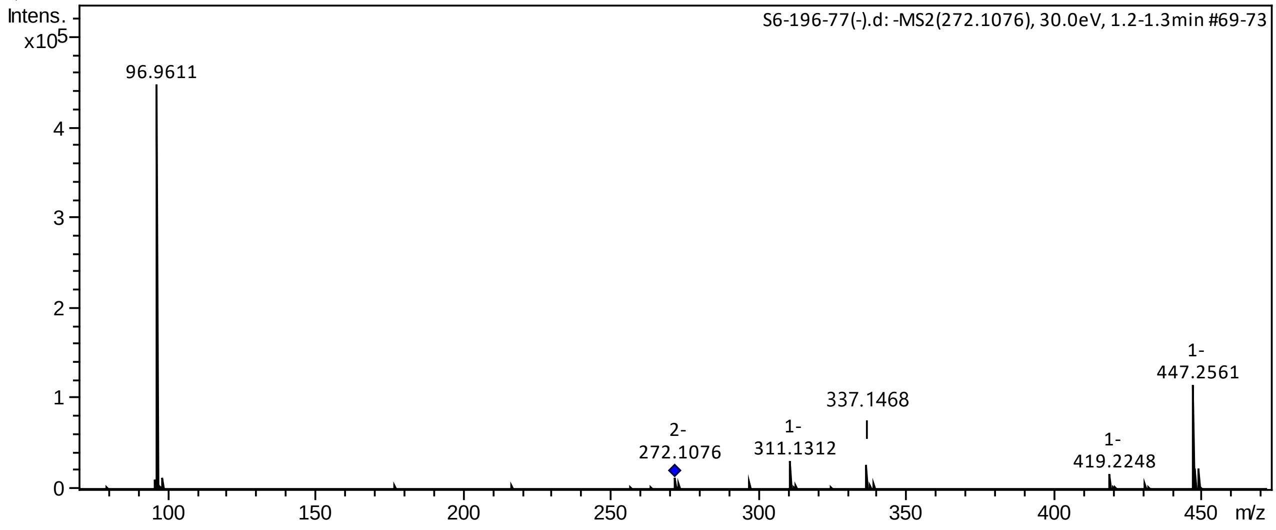

Figure S2. ^{1}H NMR (700.13 MHz, CD_3OD) spectrum of compound **1**.

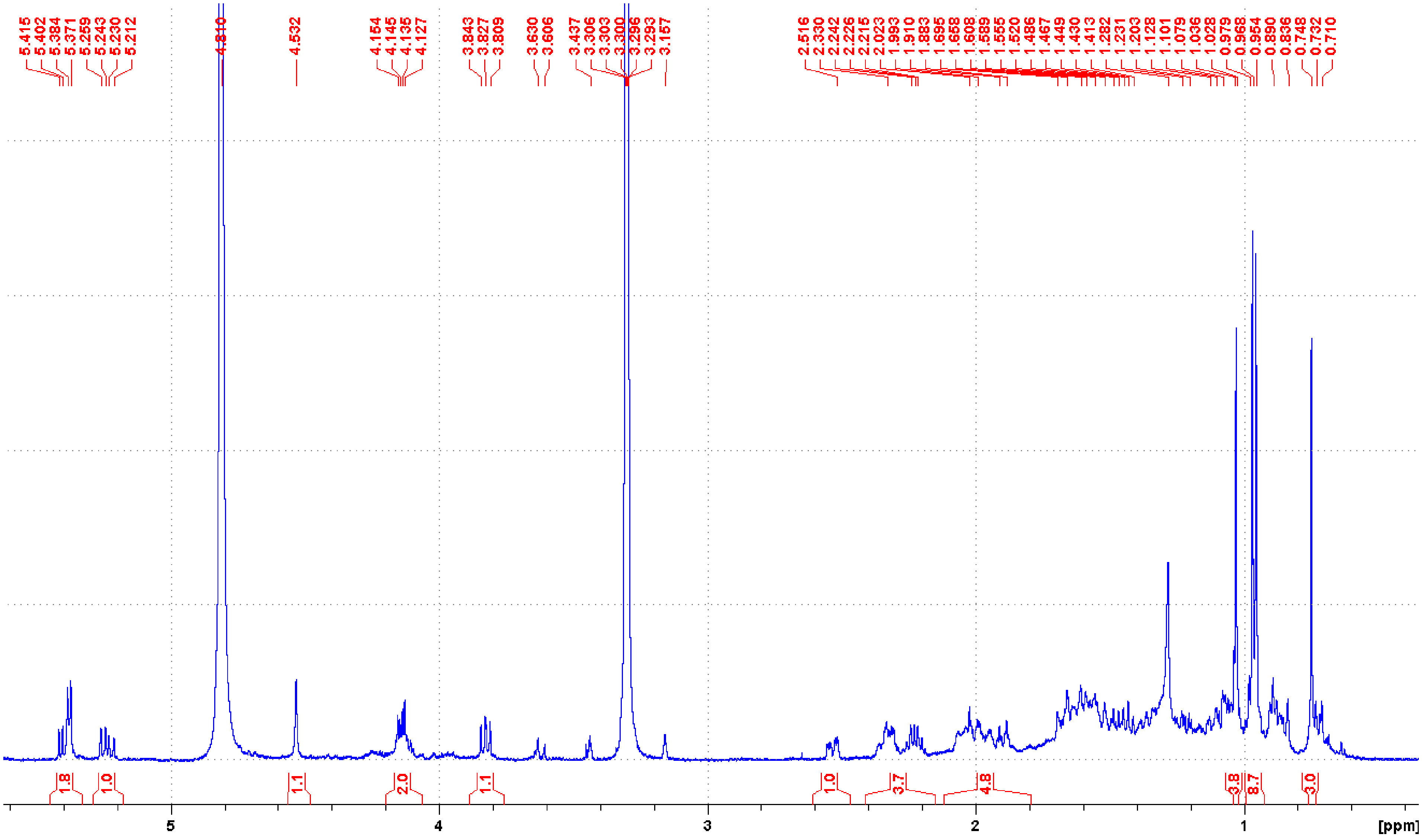

Figure S3. ^{13}C NMR (176.04 MHz, CD_3OD) spectrum of compound **1**.

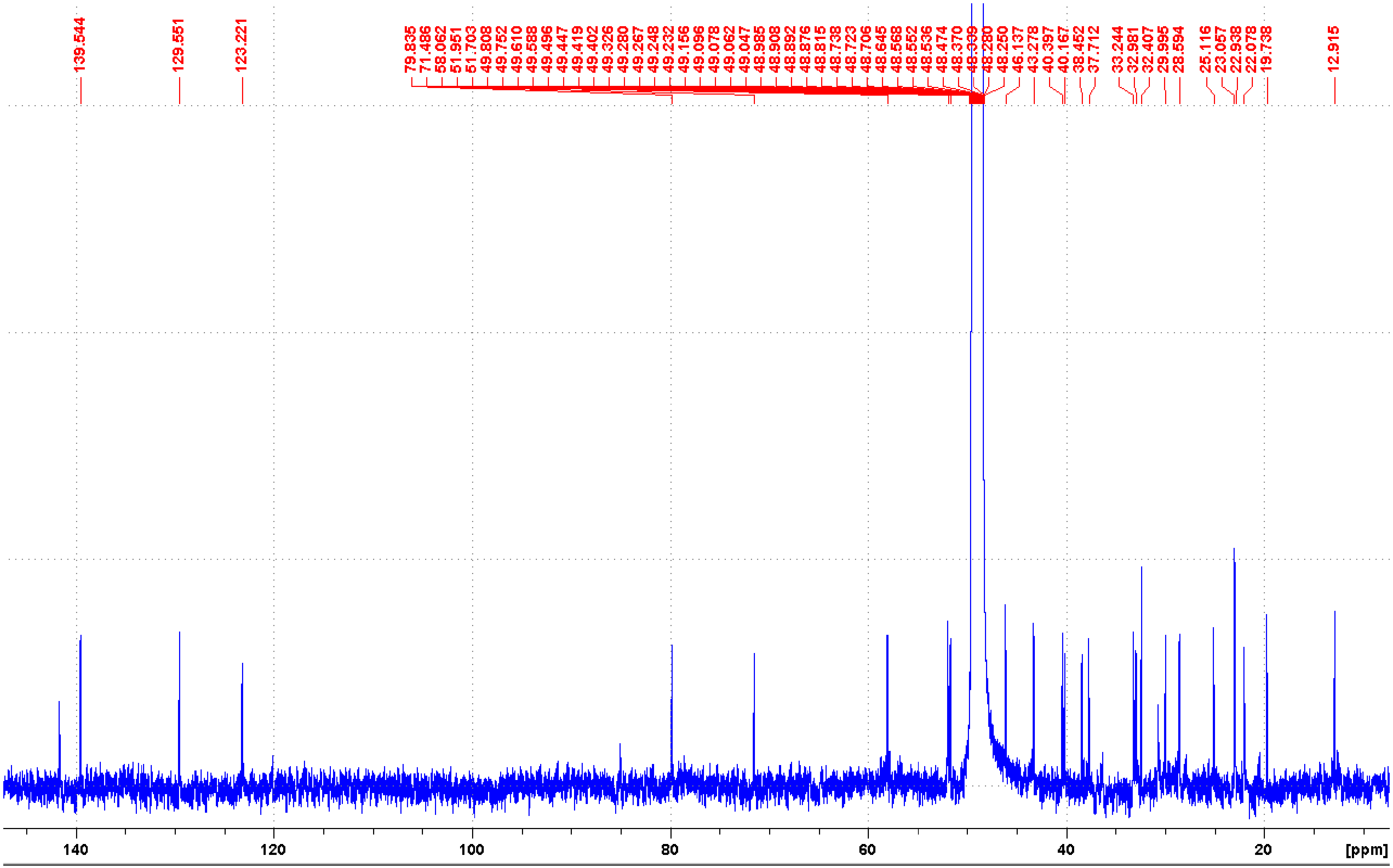

Figure S4. ^{1}H-^{1}H COSY (700.13 MHz, CD_3OD) spectrum of compound **1**.

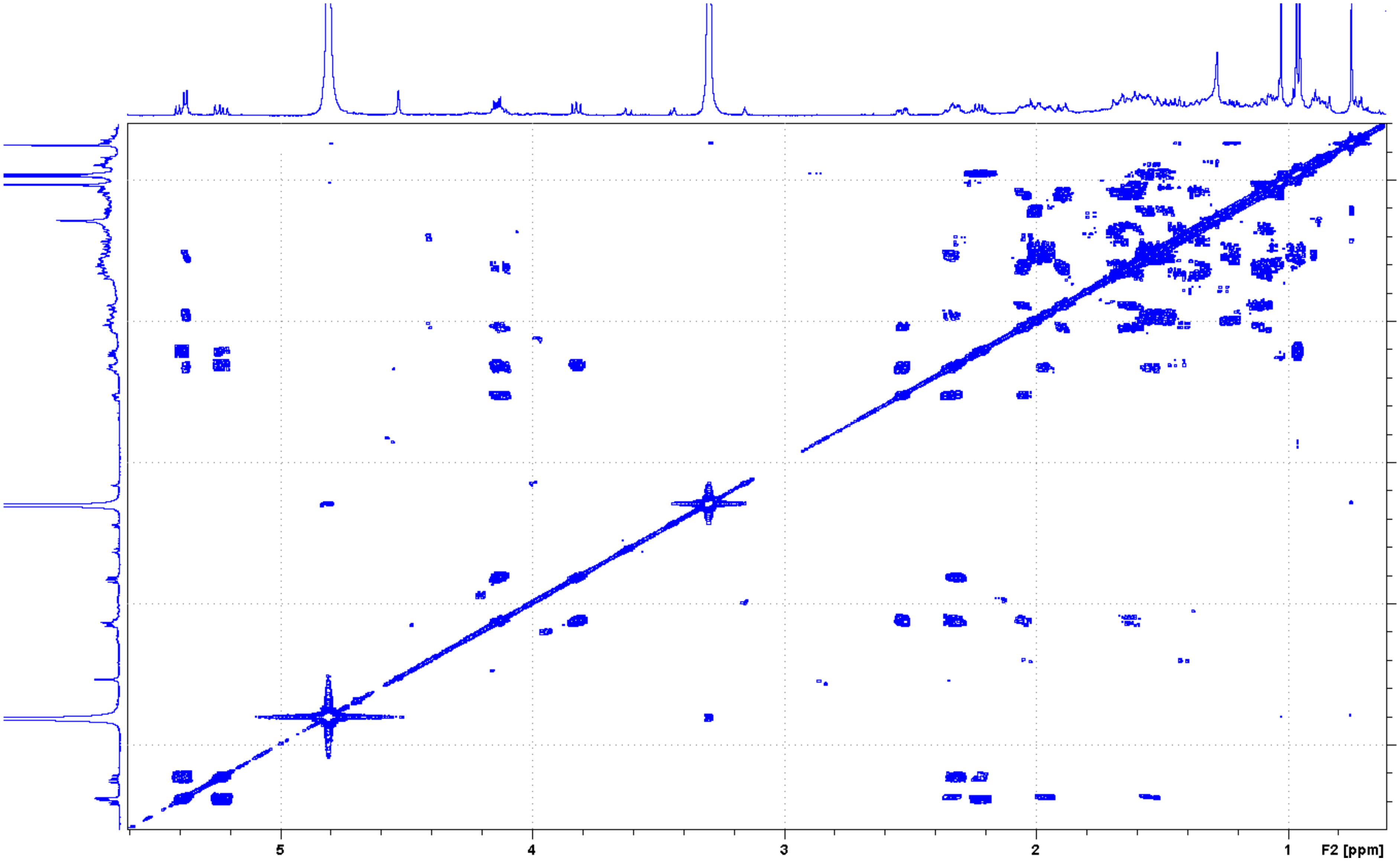

Figure S5. HSQC (700.13 MHz, CD_3OD) spectrum of compound **1**.

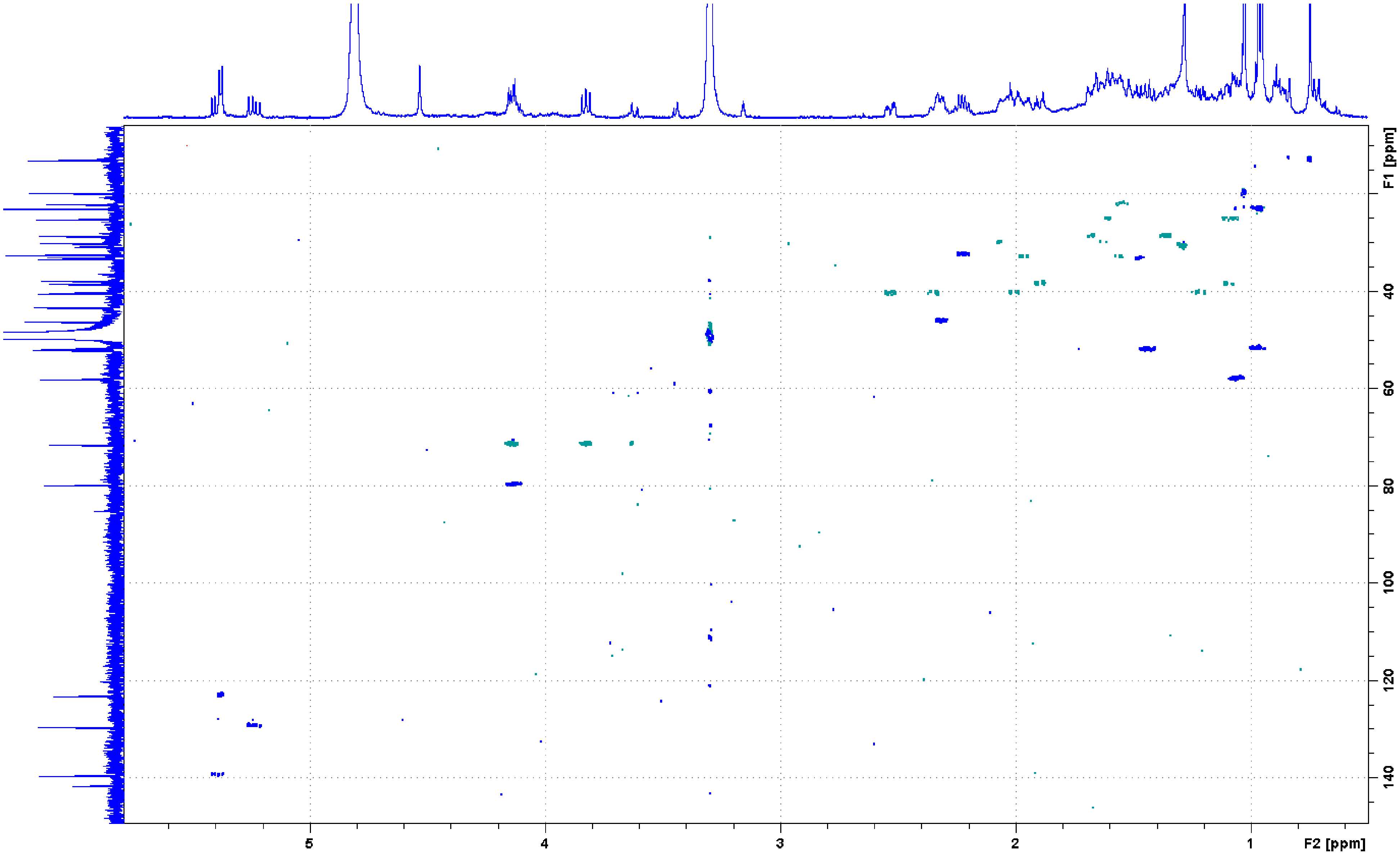

Figure S6. HMBC (700.13 MHz, CD_3OD) spectrum of compound **1**.

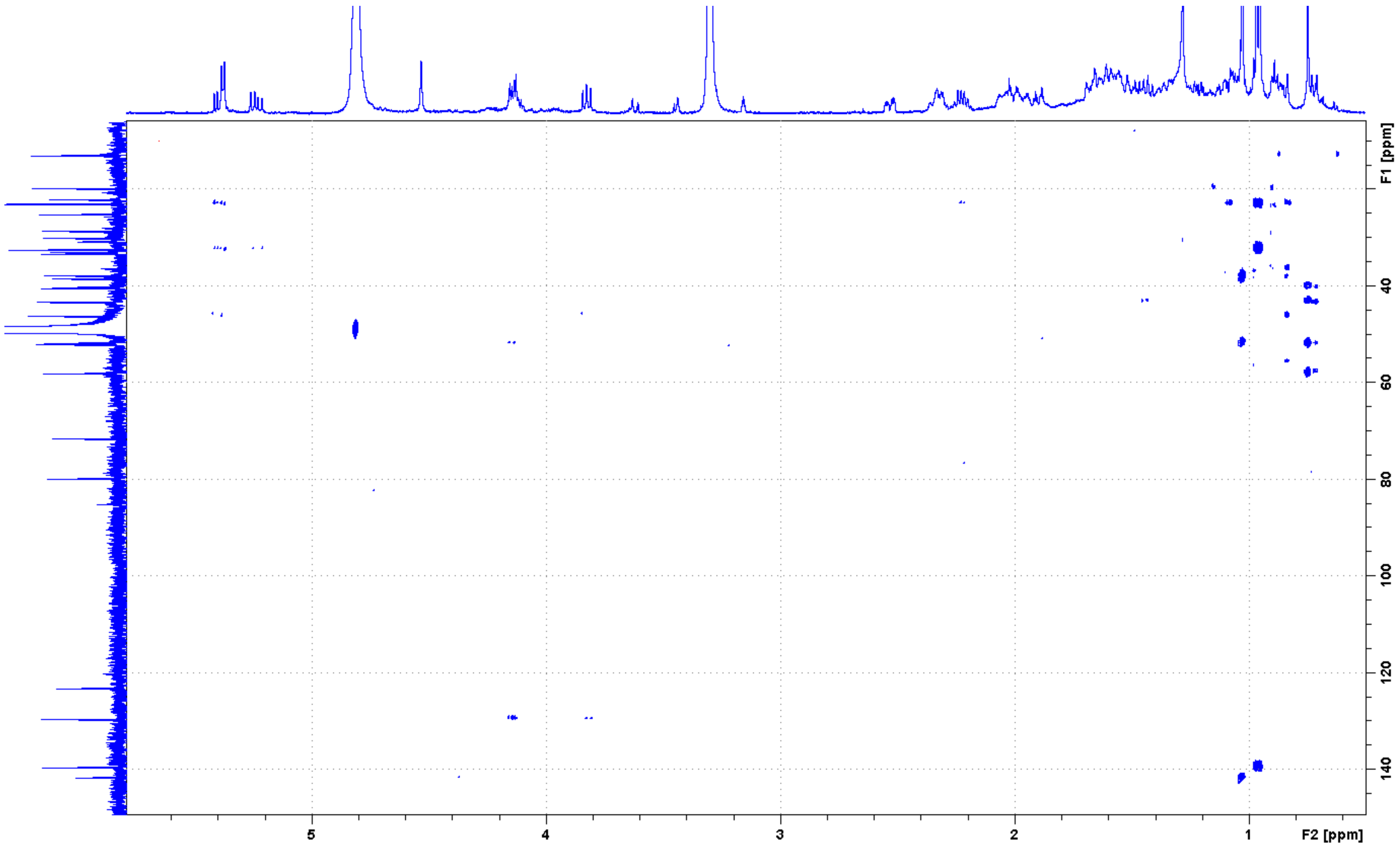

Figure S7. ROESY (700.13 MHz, CD_3OD) spectrum of compound **1**.

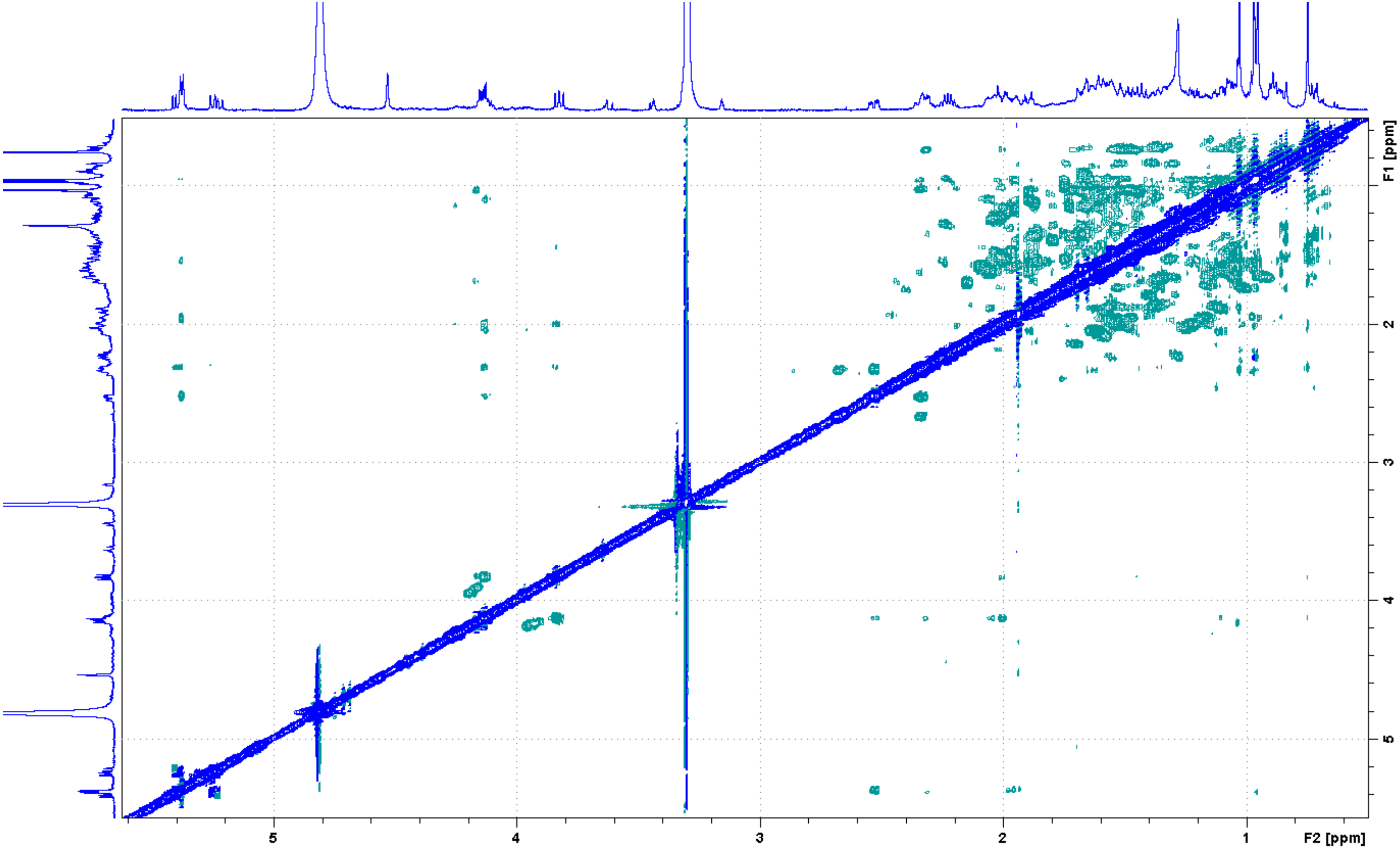

Figure S8. HRESIMS and HRESIMS/MS spectra of compound **2**.

(–)HRESIMS: [M – 2Na + H]$^{-}$ and [M – 2Na]$^{2-}$ ions

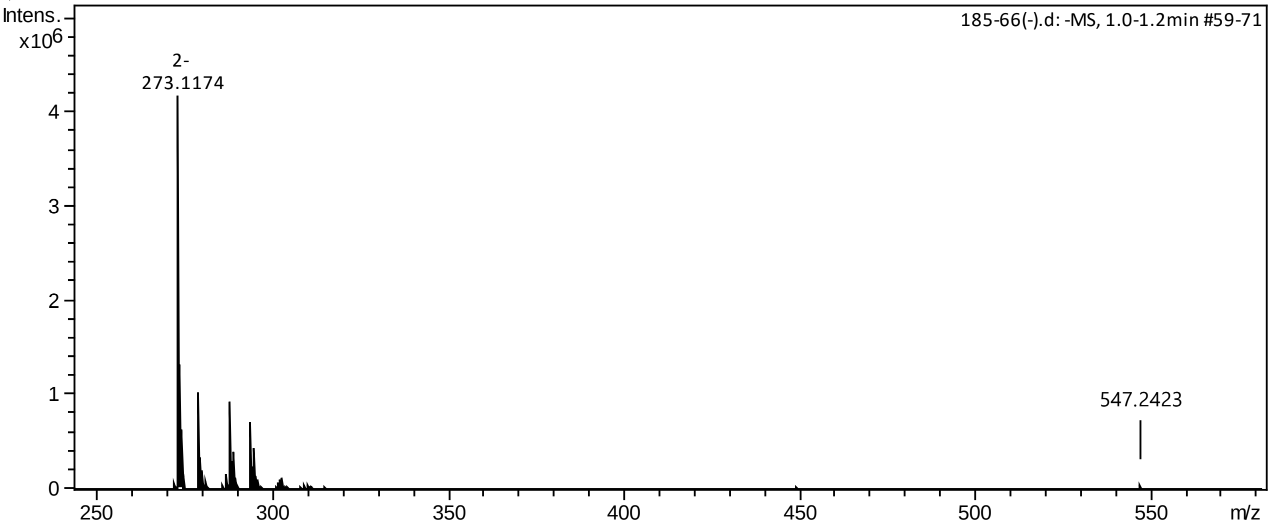

(+)HRESIMS: [M + Na]$^{+}$ and [M + H]$^{+}$ ions

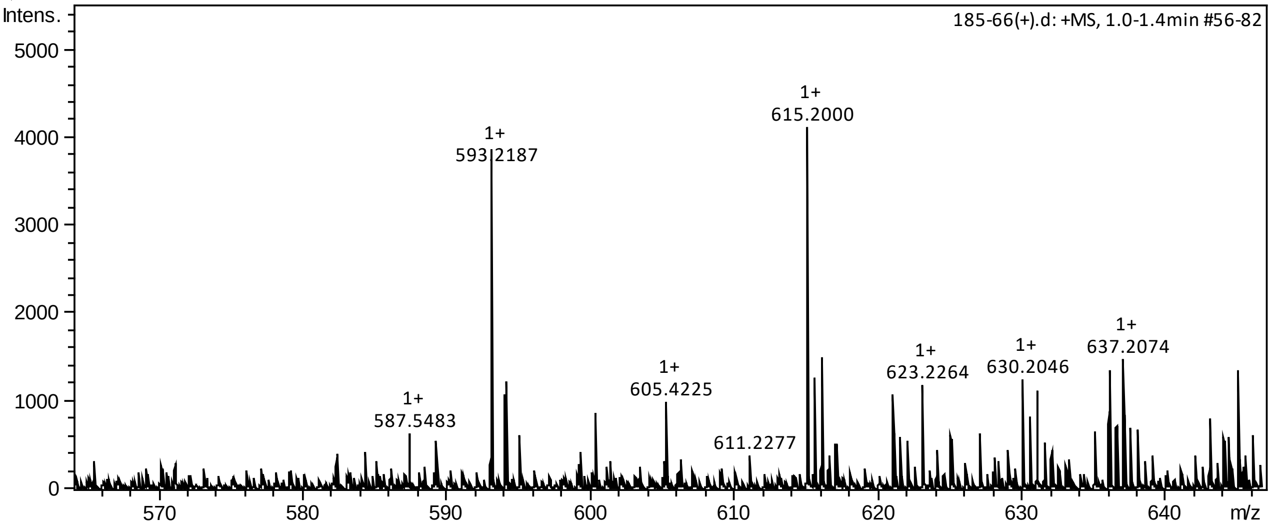

(–)HRESIMS/MS of the [M – 2Na]$^{2-}$ ion at *m/z* 273.1171

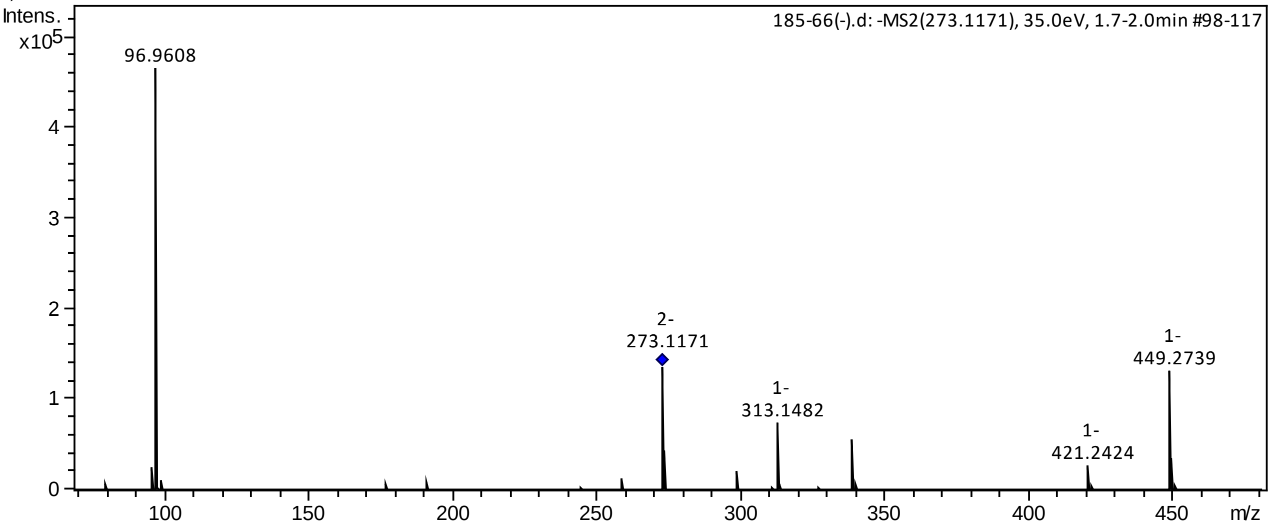

Figure S9. ^{1}H NMR (700.13 MHz, CD_3OD) spectrum of compound **2**.

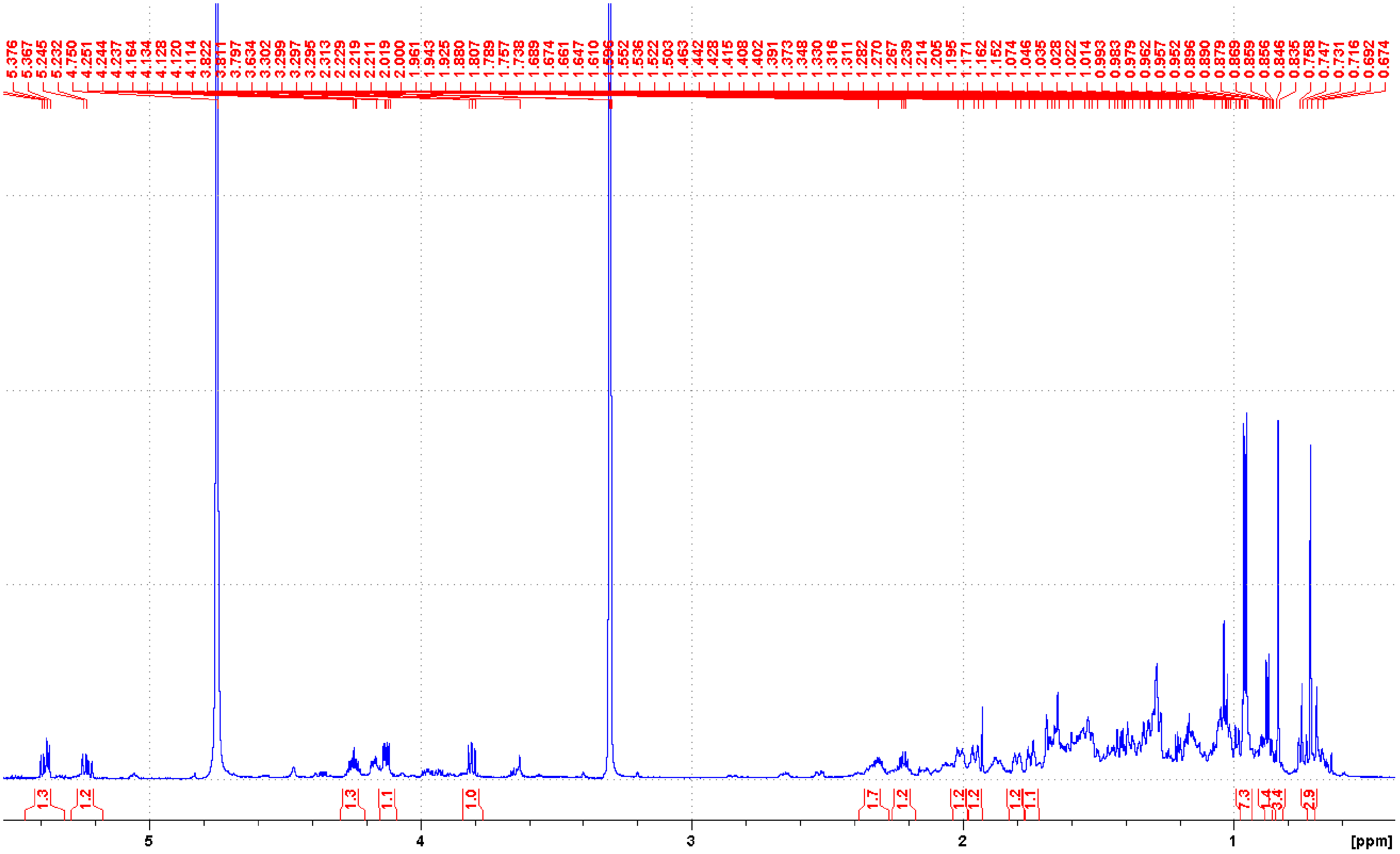

Figure S10. ^{13}C NMR (176.04 MHz, CD_3OD) spectrum of compound **2**.

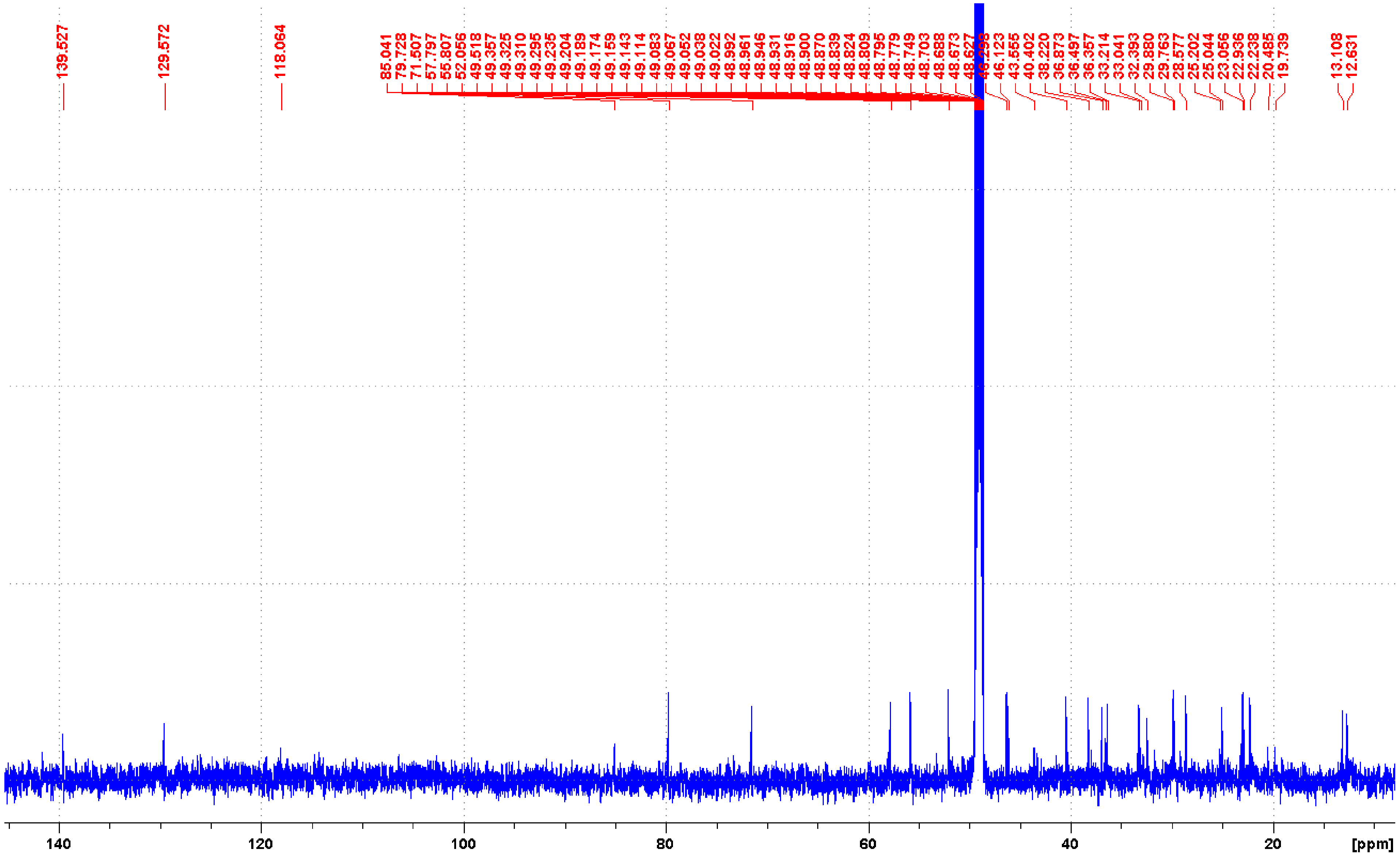

Figure S11. ^{1}H-^{1}H COSY (700.13 MHz, CD_3OD) spectrum of compound **2**.

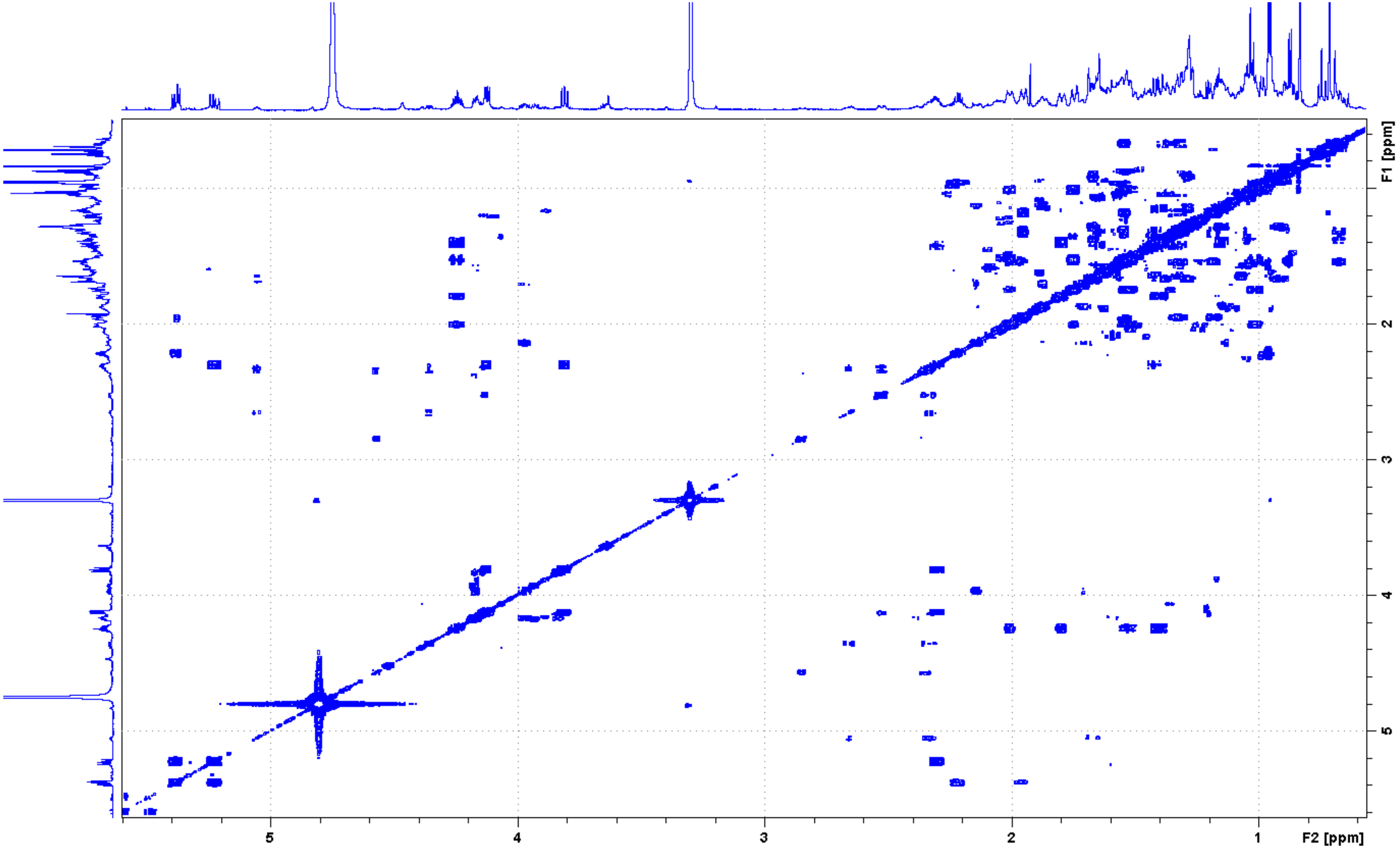

Figure S12. HSQC (700.13 MHz, CD_3OD) spectrum of compound **2**.

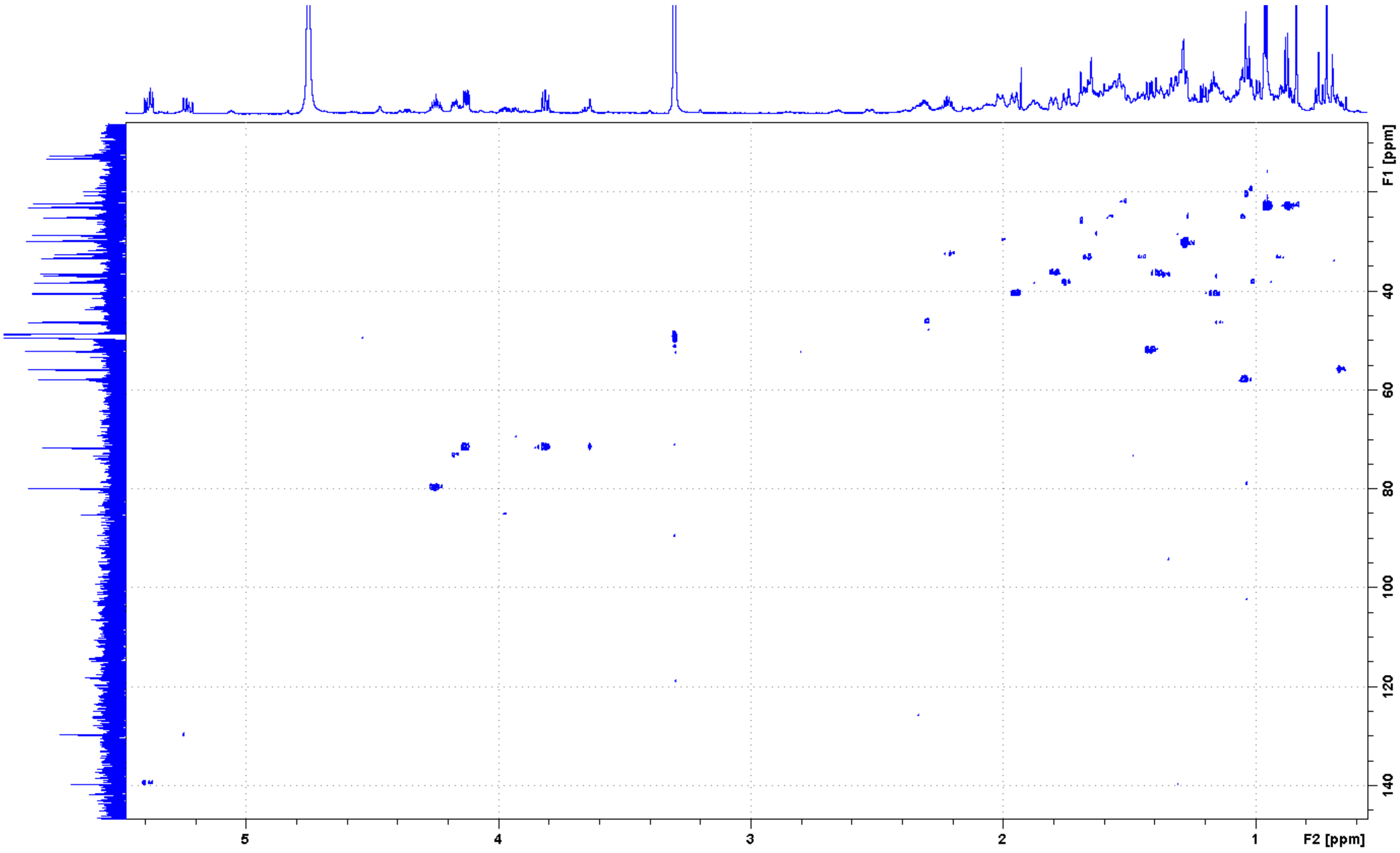

Figure S13. HMBC (700.13 MHz, CD_3OD) spectrum of compound **2**.

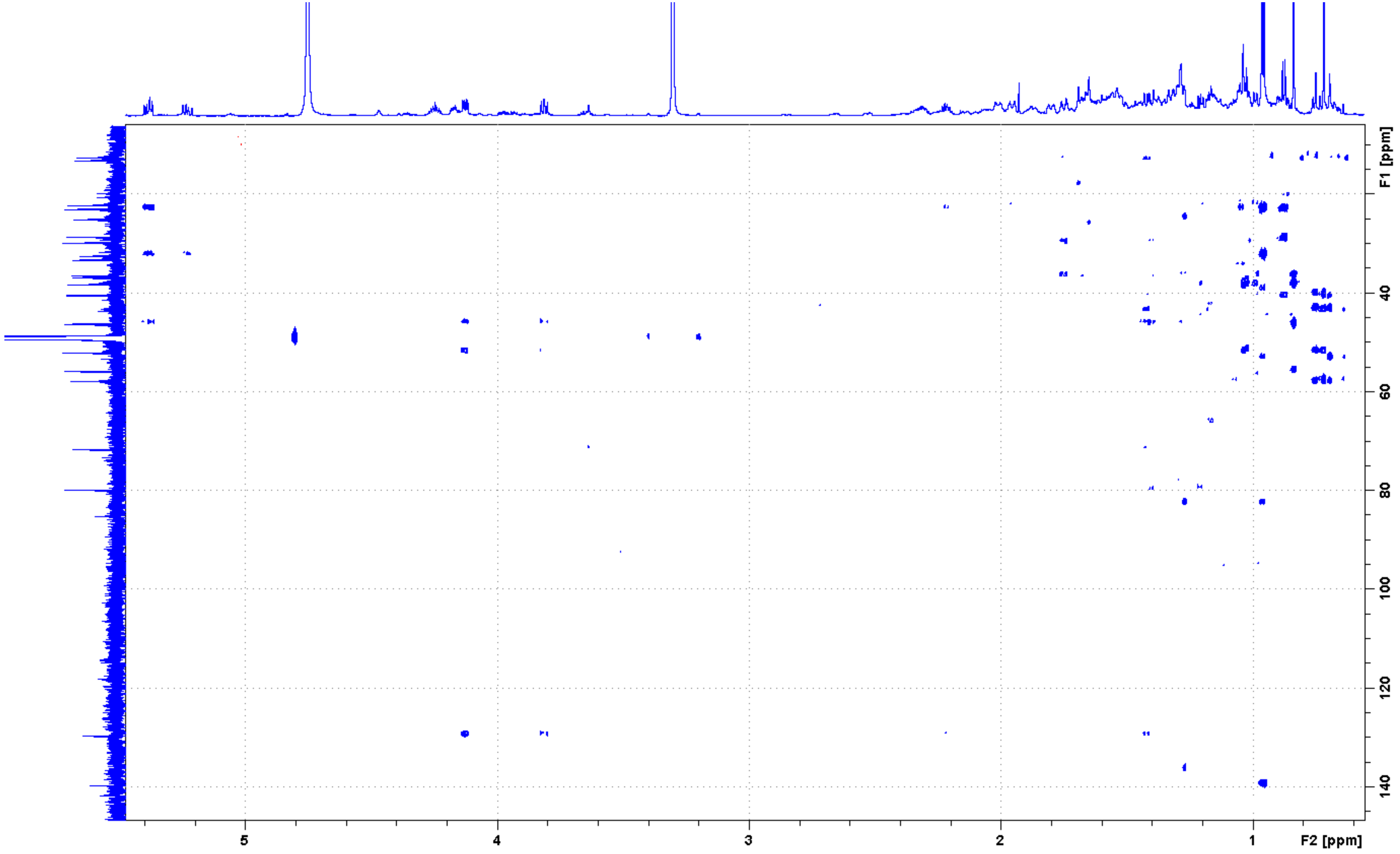

Figure S14. ROESY (700.13 MHz, CD_3OD) spectrum of compound **2**.

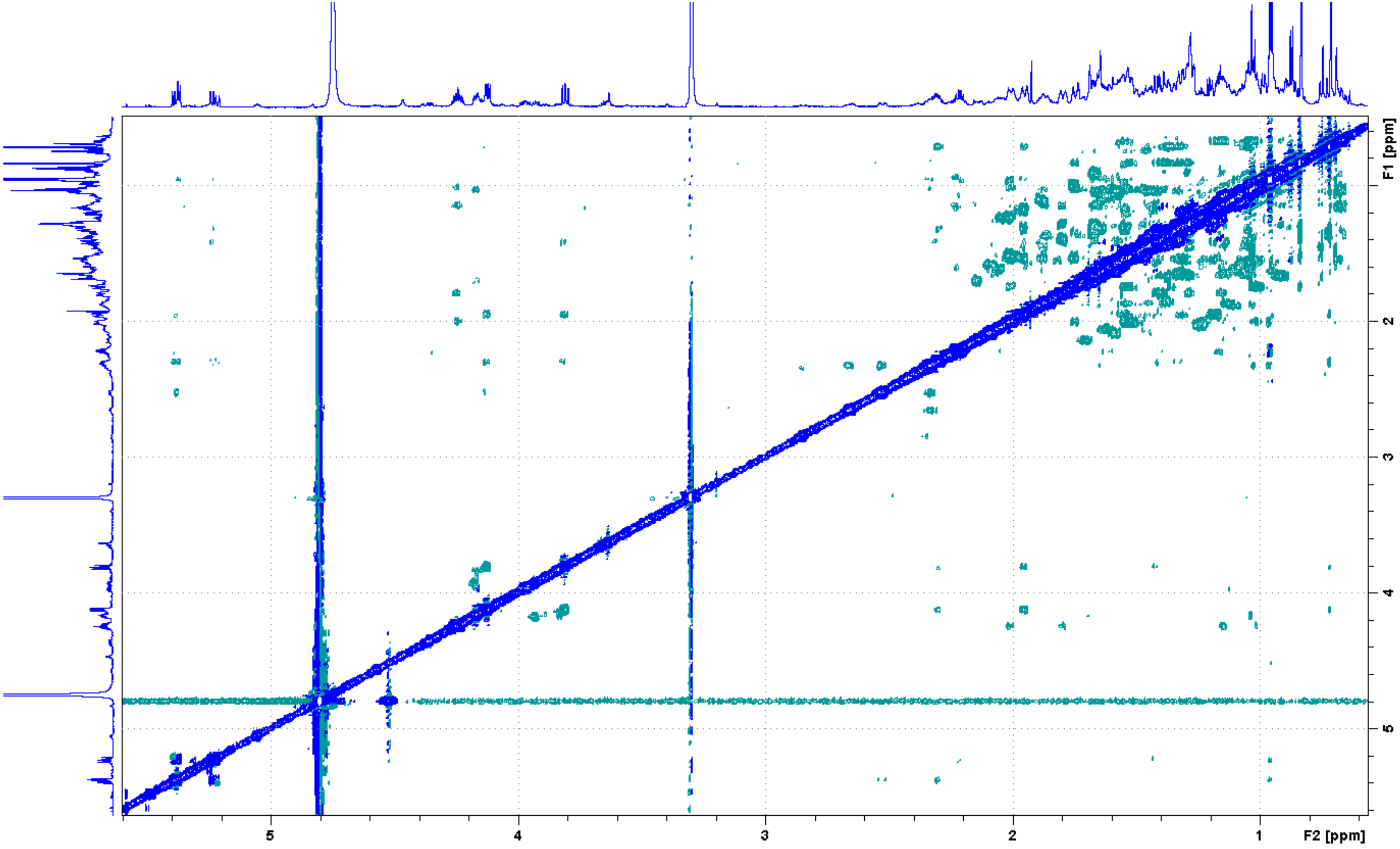

Figure S15. HRESIMS and HRESIMS/MS spectra of compound **3**.

(–)HRESIMS: [M – Na]$^{-}$ and [M – 2Na]$^{2-}$ ions

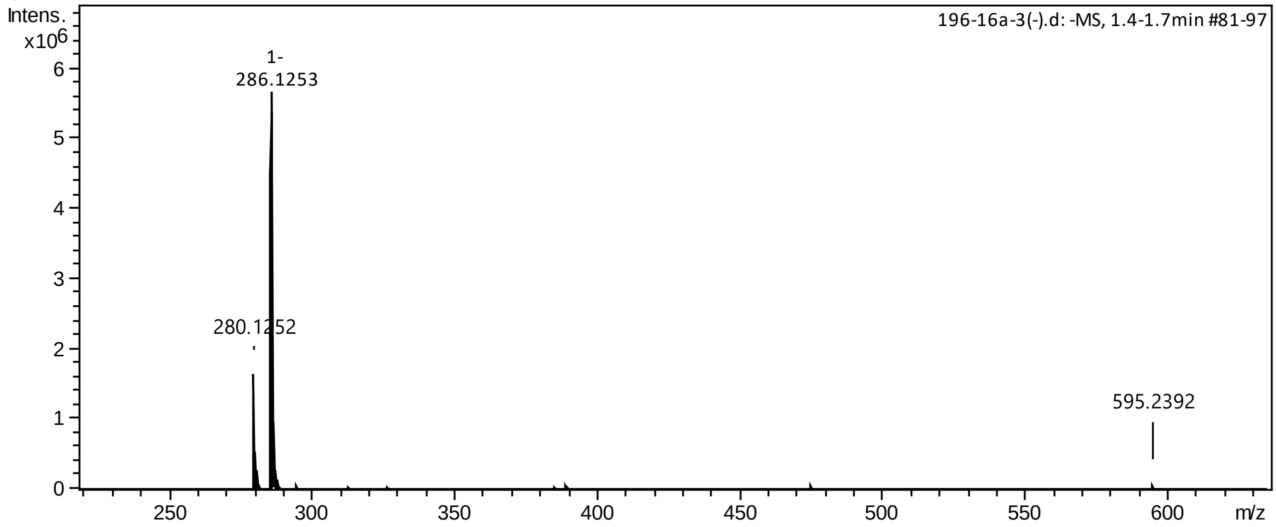

(+)HRESIMS: [M + Na]$^{+}$ ion

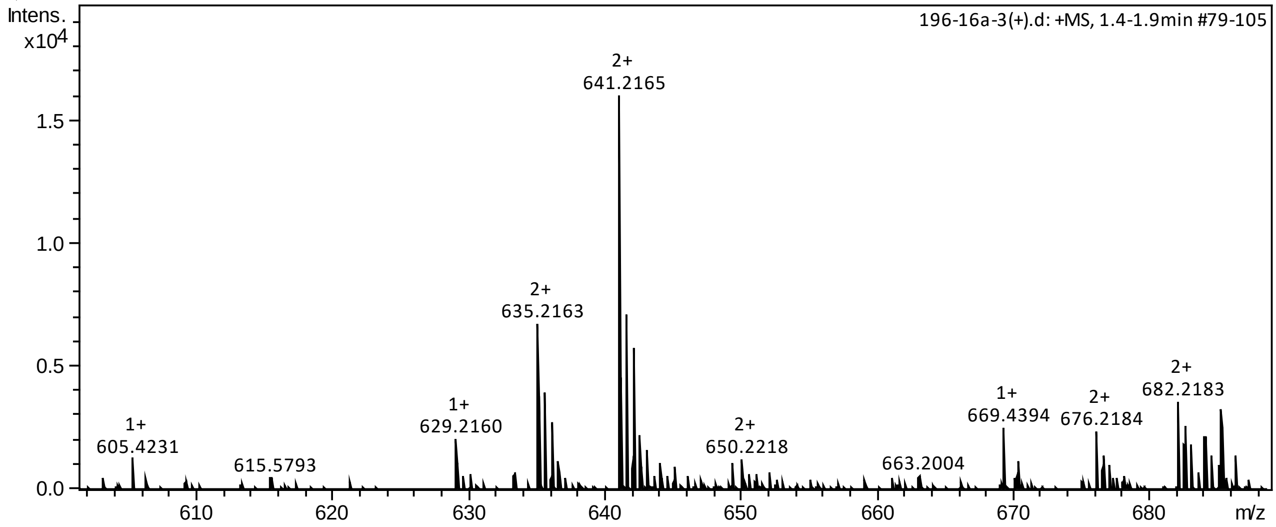

(–)HRESIMS/MS of the [M – 2Na]$^{2-}$ ion at *m/z* 286.1254

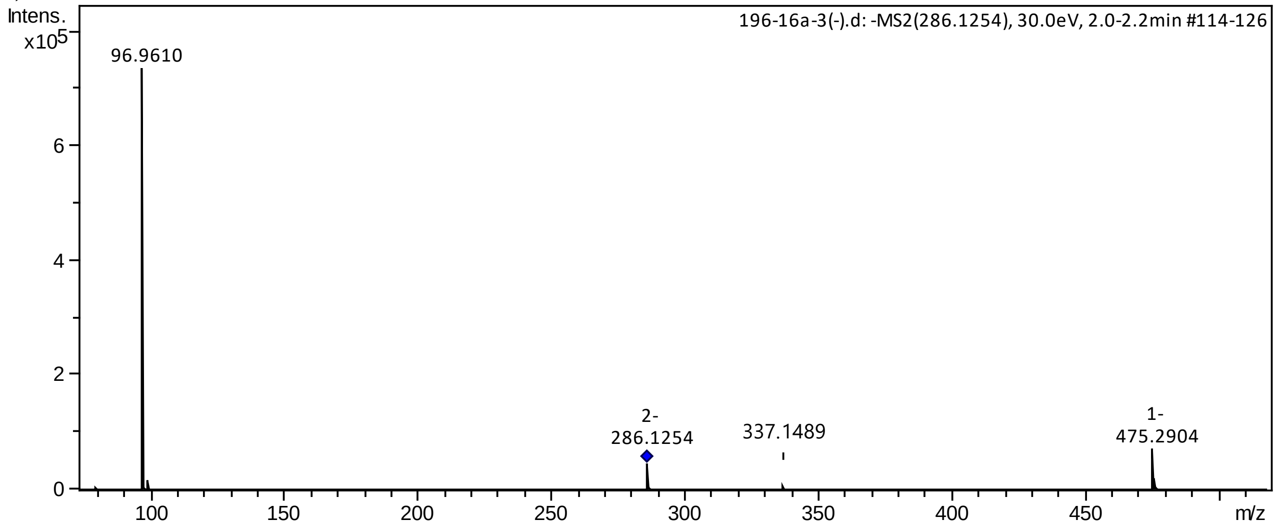

Figure S16. ^{1}H NMR (700.13 MHz, CD_3OD) spectrum of compound **3**.

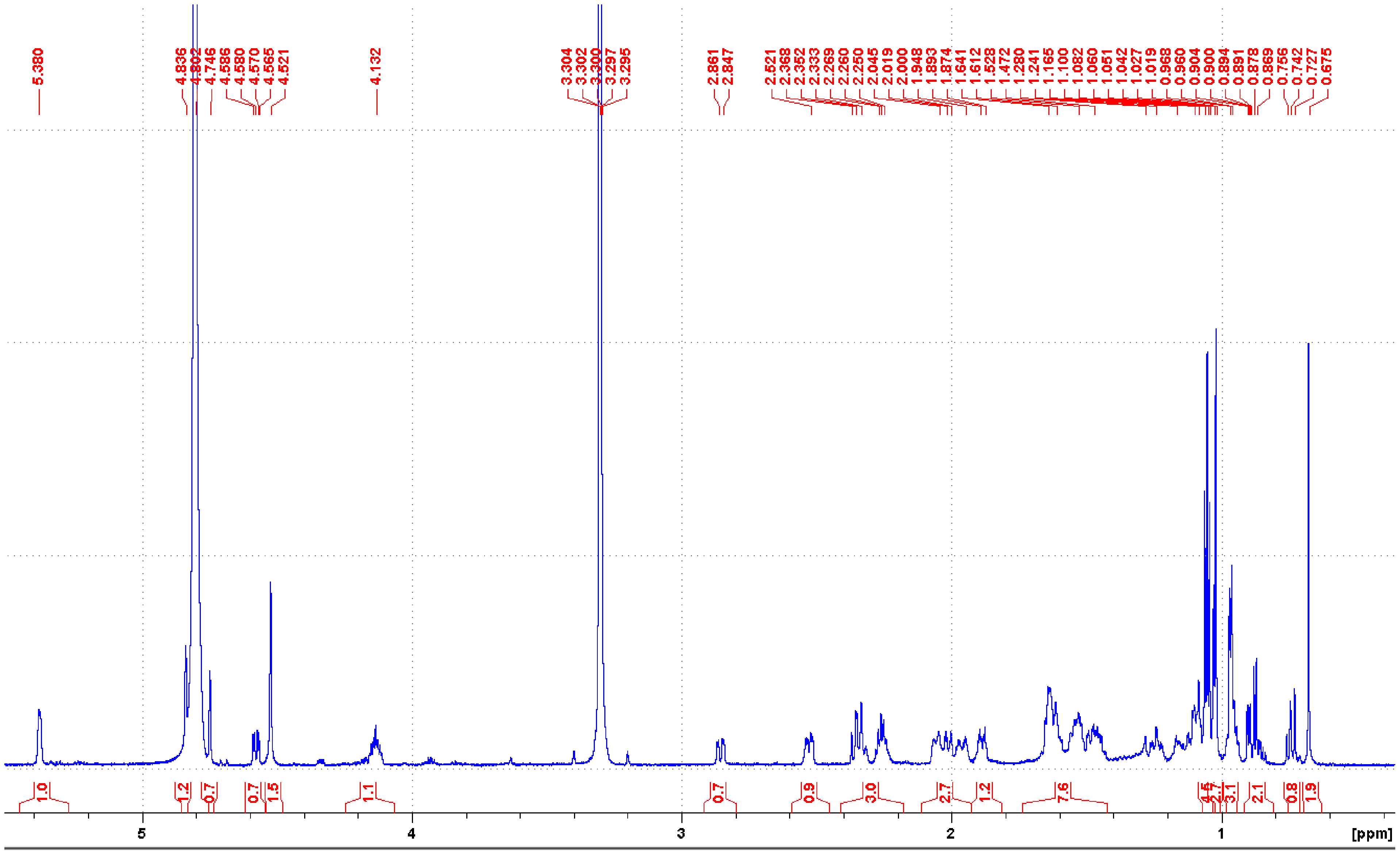

Figure S17. ^{13}C NMR (176.04 MHz, CD_3OD) spectrum of compound **3**.

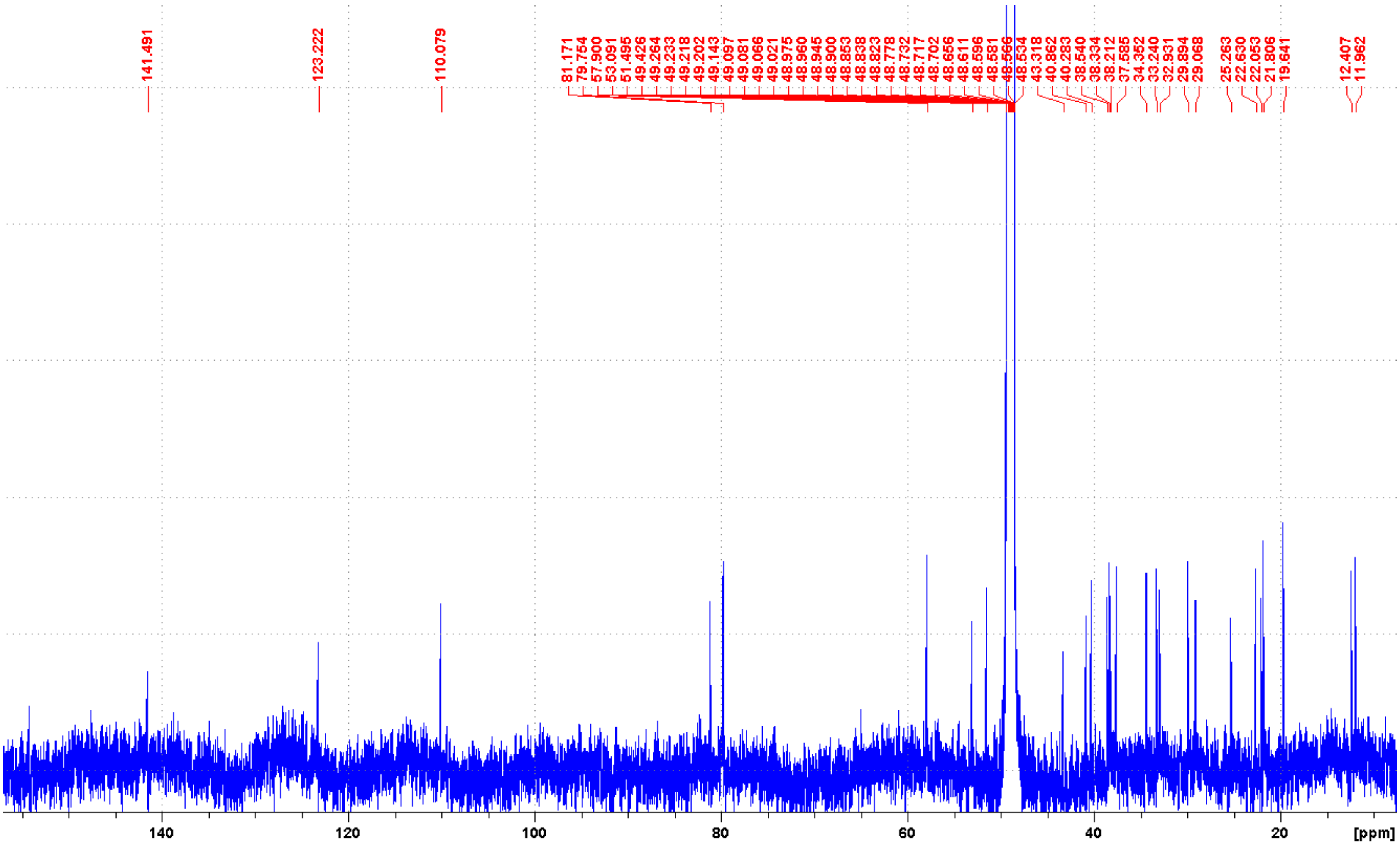

Figure S18. ^{1}H-^{1}H COSY (700.13 MHz, CD_3OD) spectrum of compound **3**.

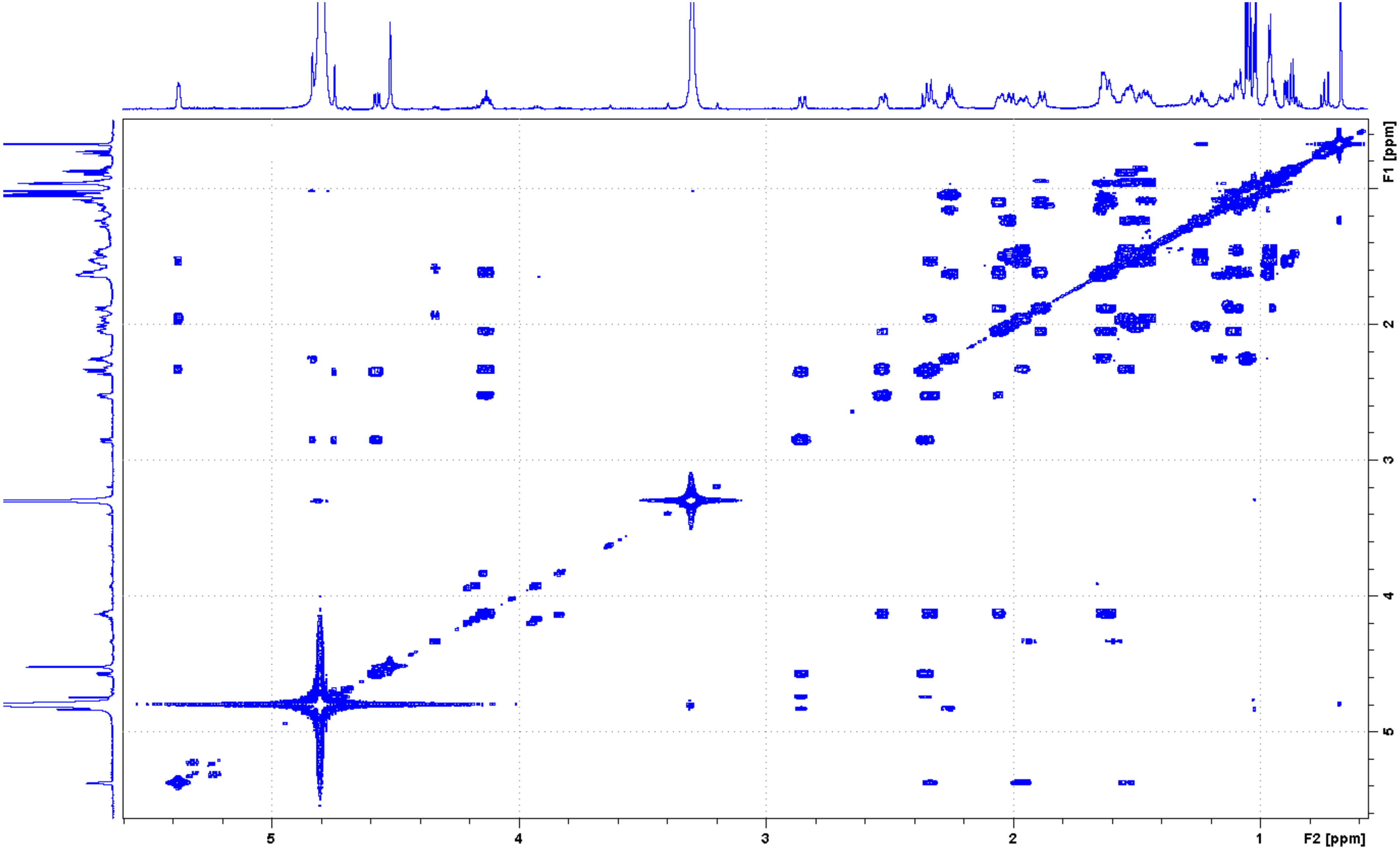

Figure S19. HSQC (700.13 MHz, CD_3OD) spectrum of compound **3**.

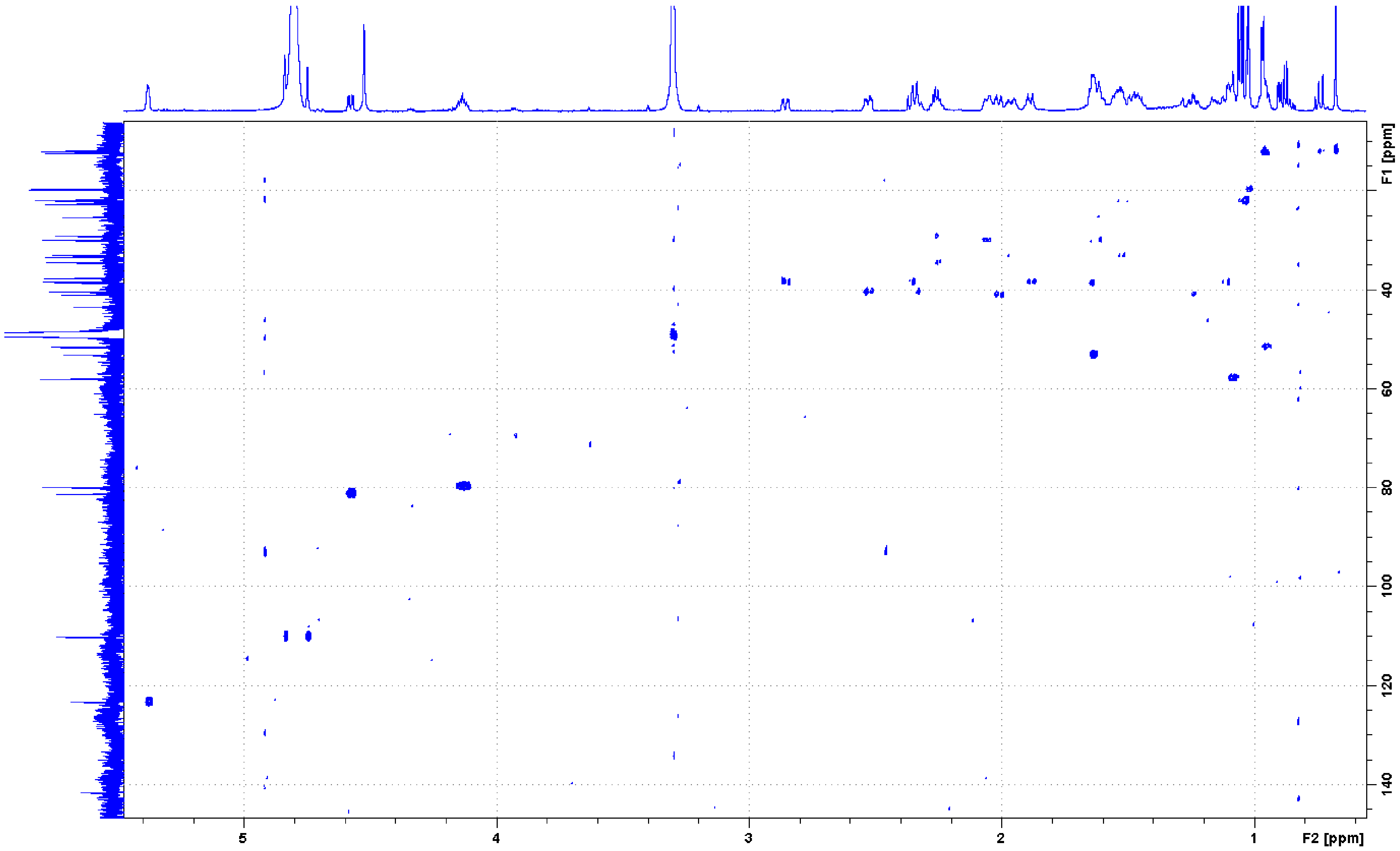

Figure S20. HMBC (700.13 MHz, CD_3OD) spectrum of compound **3**.

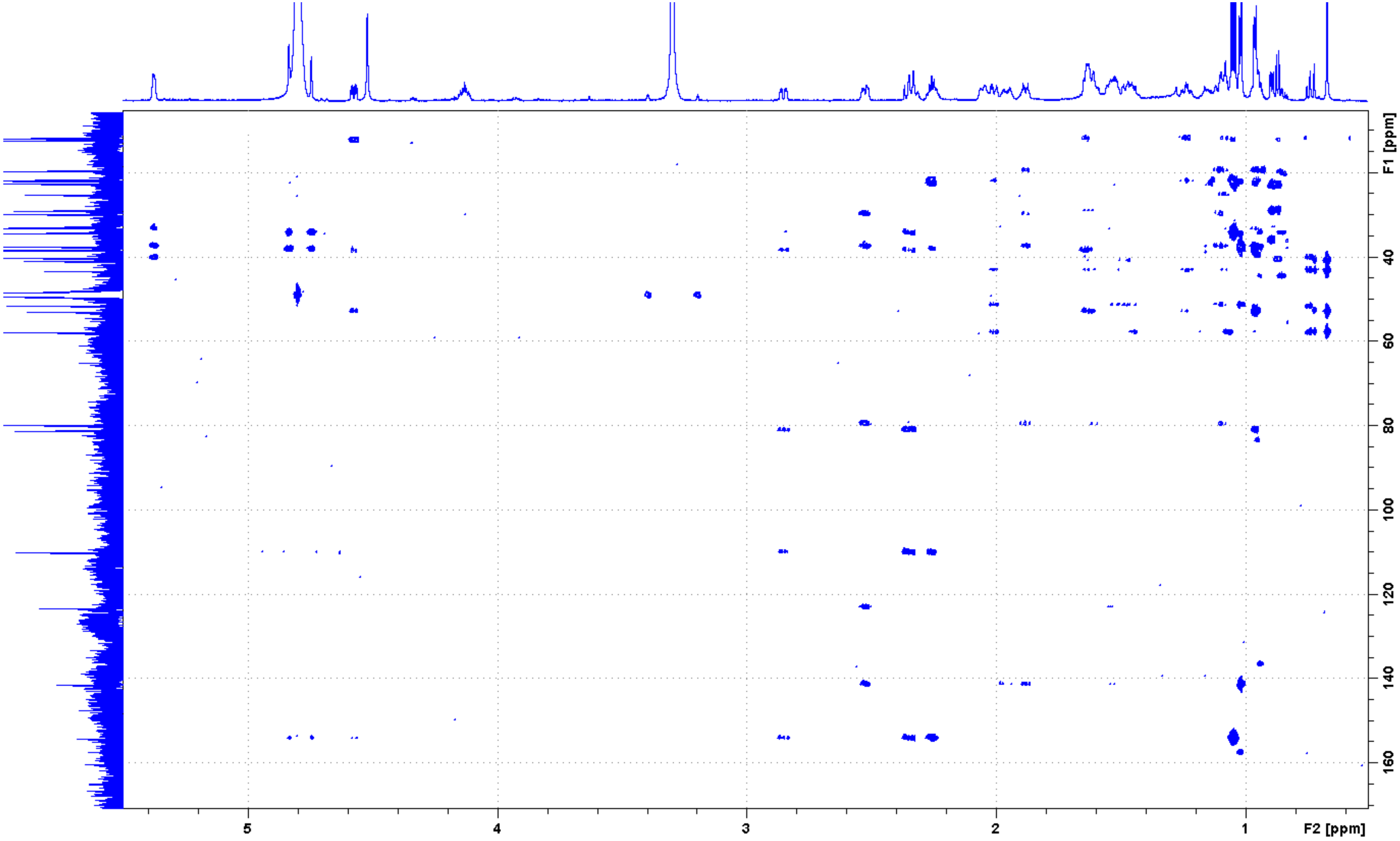

Figure S21. ROESY (700.13 MHz, CD_3OD) spectrum of compound **3**.

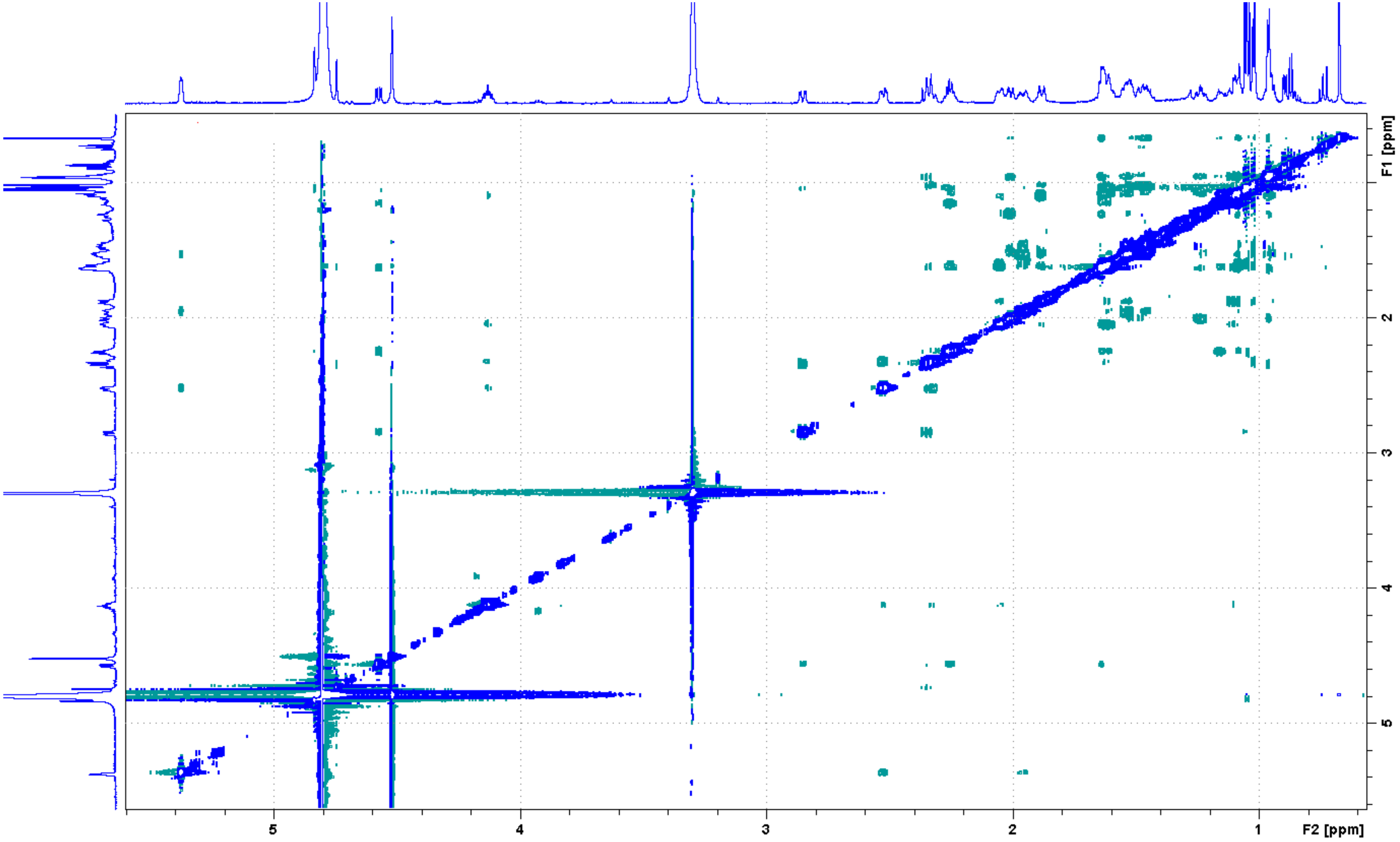

Figure S22. HRESIMS and HRESIMS/MS spectra of compound **4**

(–)HRESIMS: [M – Na]$^{–}$ and [M – 2Na]$^{2–}$ ions

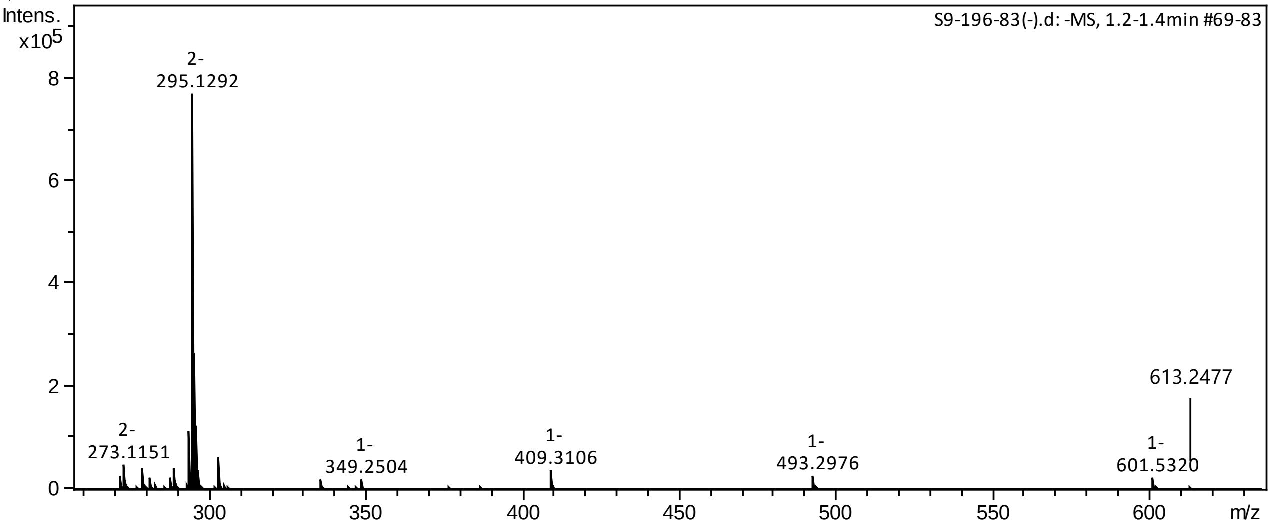

(+)HRESIMS: [M + Na]$^{+}$ ion

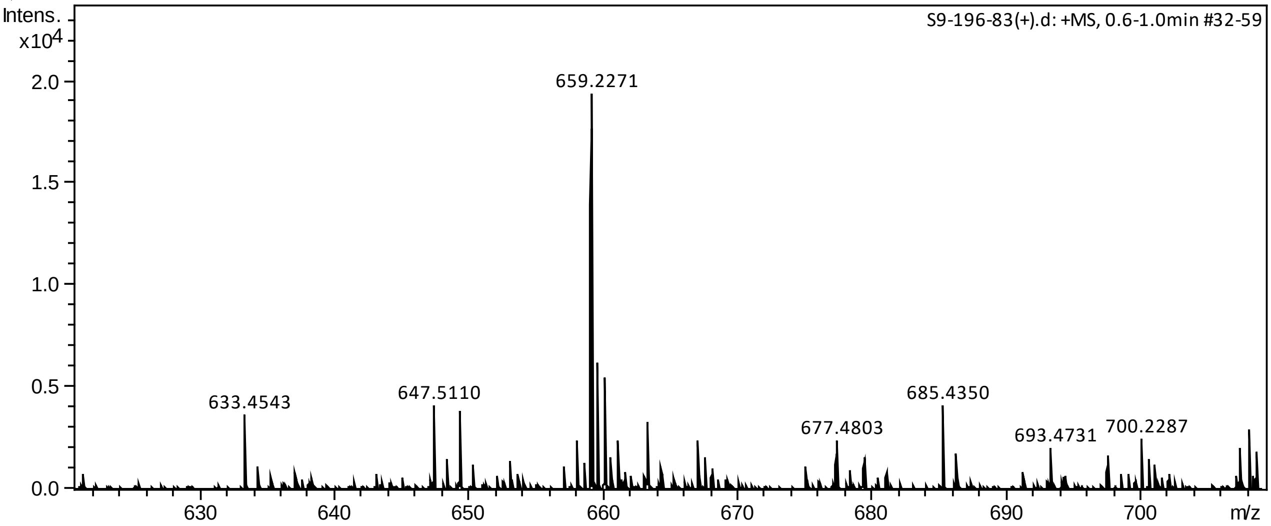

(–)HRESIMS/MS of the [M – 2Na]$^{2–}$ ion at *m/z* 295.1280

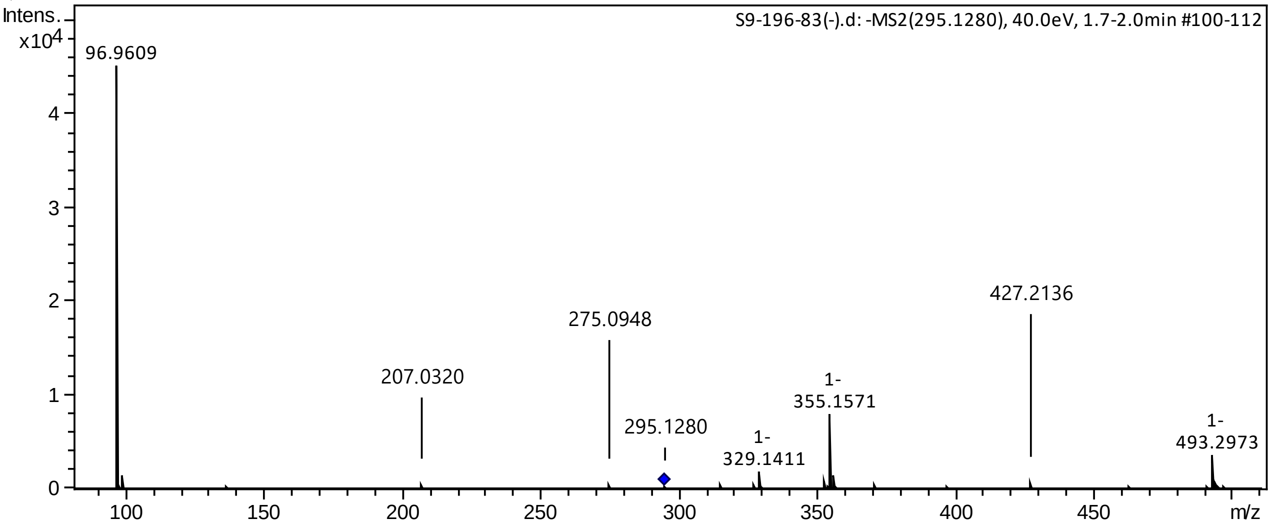

Figure S23. ^{1}H NMR (700.13 MHz, CD_3OD) spectrum of compound **4**.

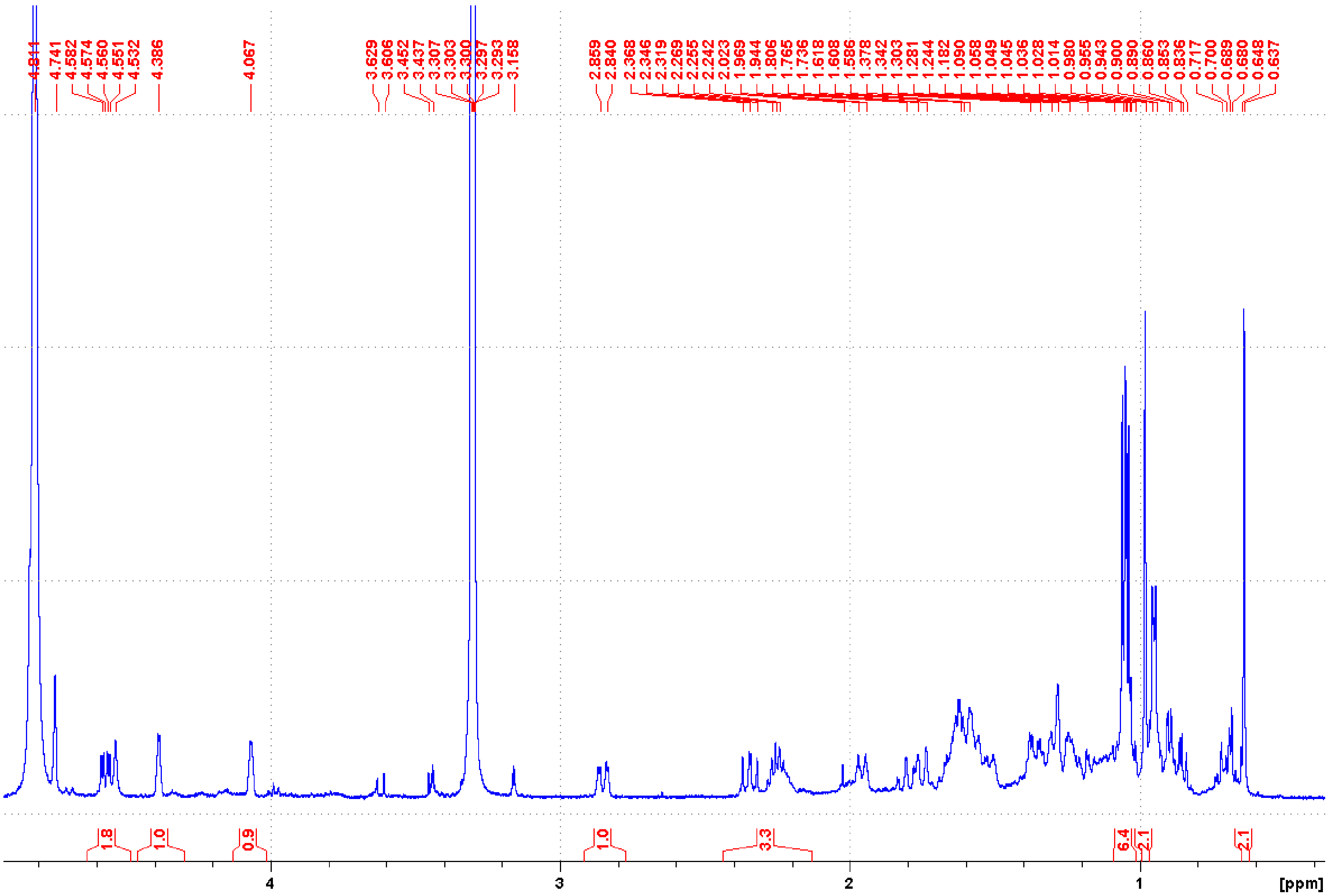

Figure S24. ^{13}C NMR (176.04 MHz, CD_3OD) spectrum of compound **4**.

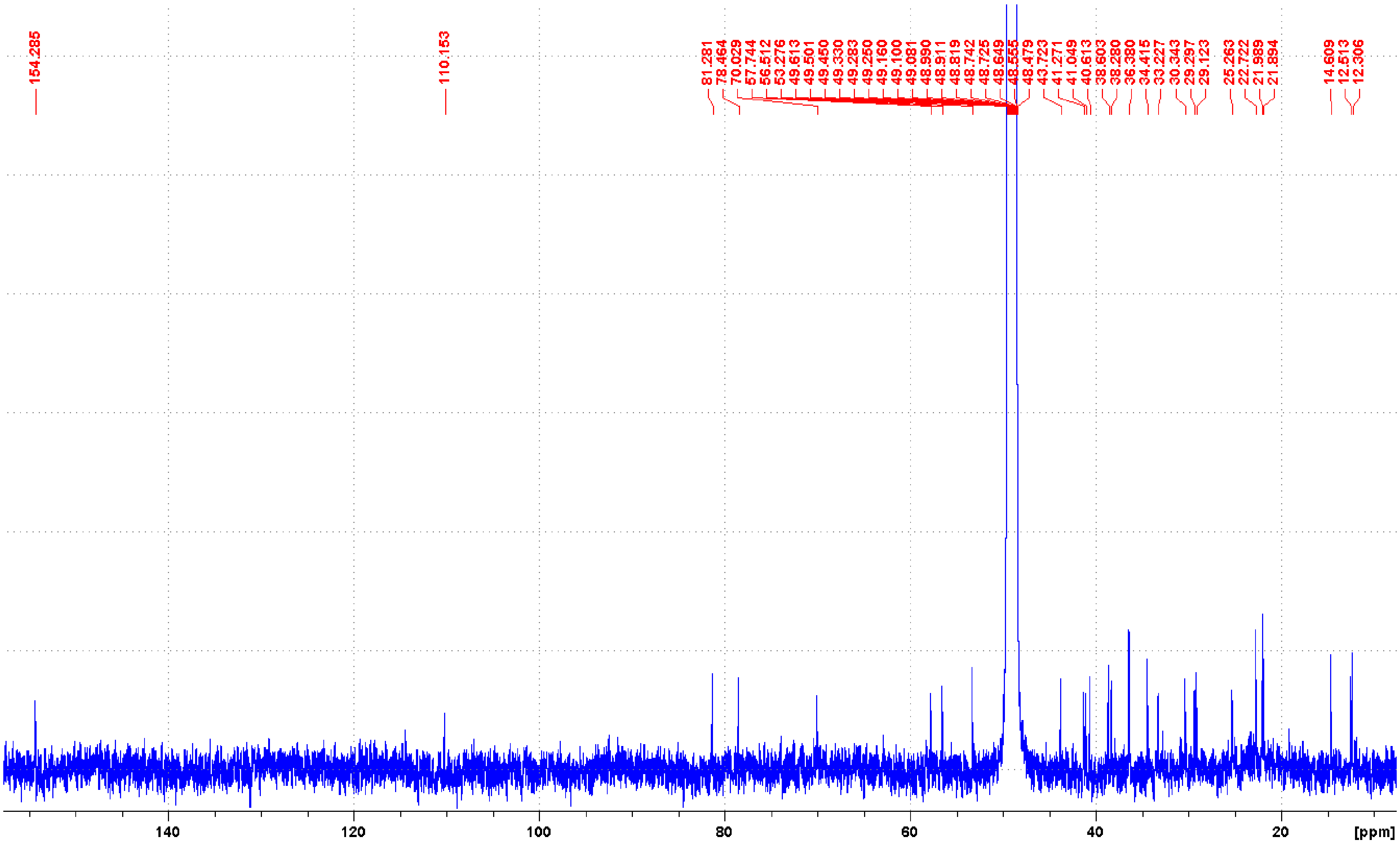

Figure S25. ^{1}H-^{1}H COSY (700.13 MHz, CD_3OD) spectrum of compound **4**.

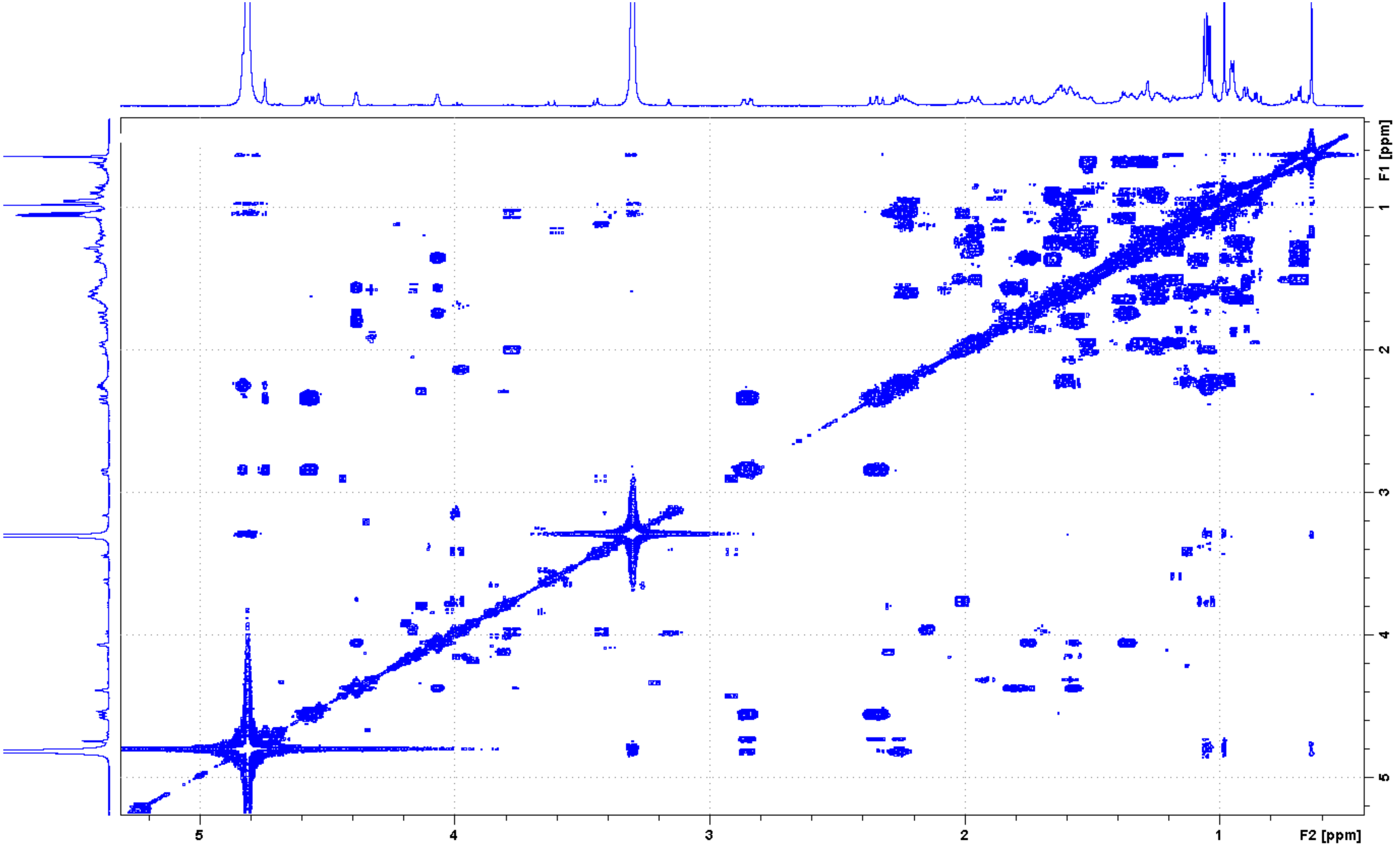

Figure S26. HSQC (700.13 MHz, CD_3OD) spectrum of compound **4**.

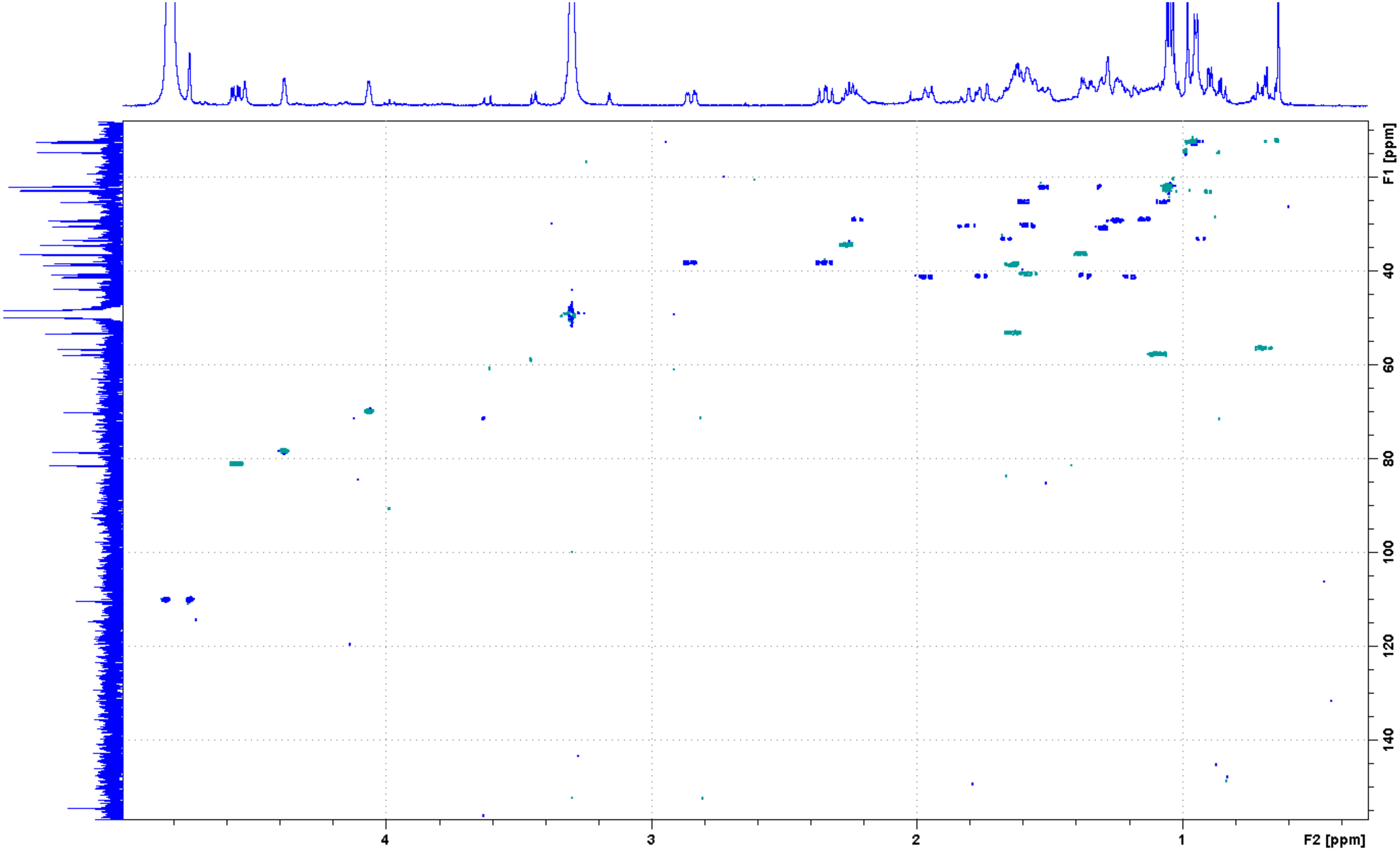

Figure S27. HMBC (700.13 MHz, CD_3OD) spectrum of compound **4**.

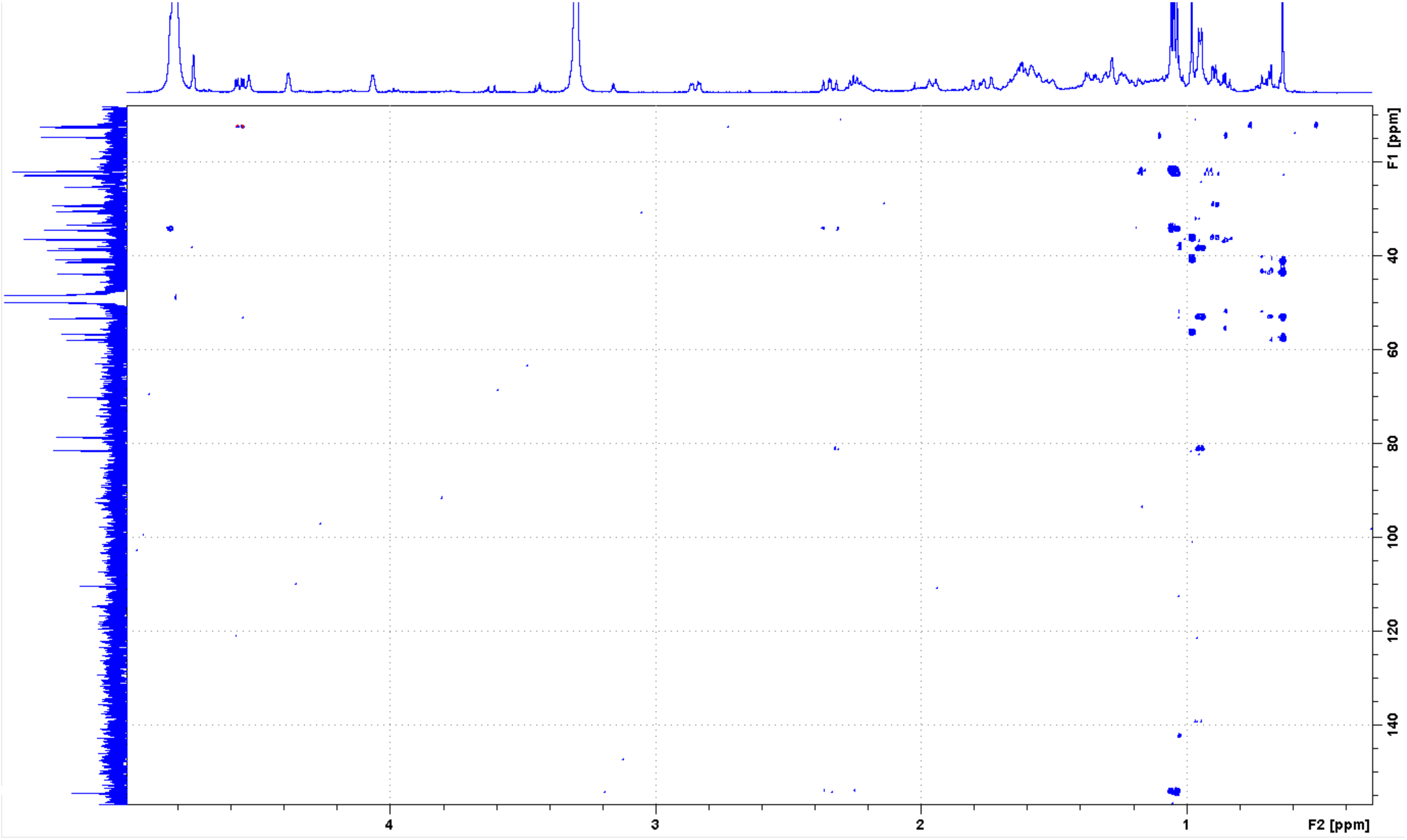

Figure S28. ROESY (700.13 MHz, CD_3OD) spectrum of compound **4**.

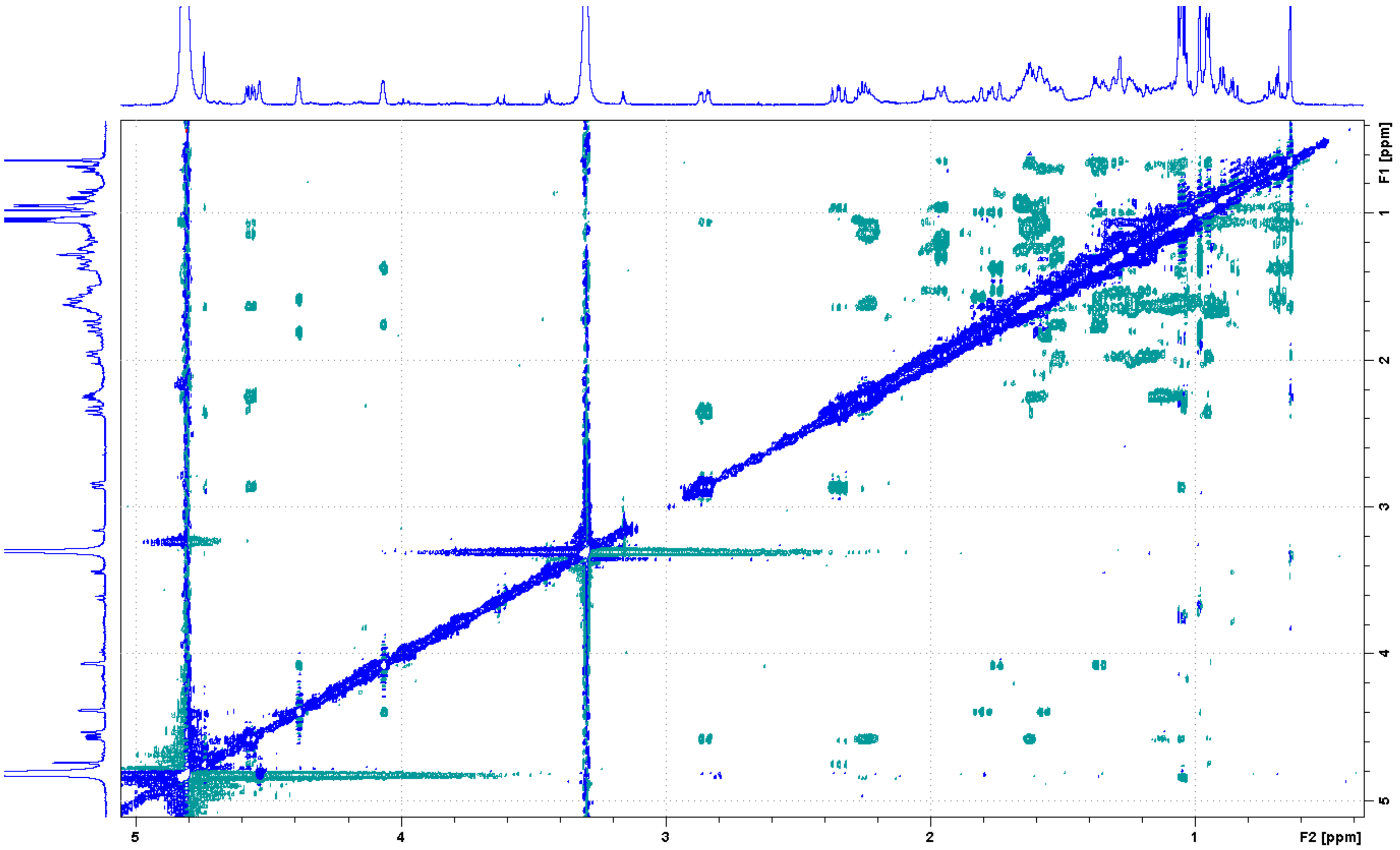

Figure S29. HRESIMS spectrum of compound **5**

(+)HRESIMS $[M + H]^+$ ion

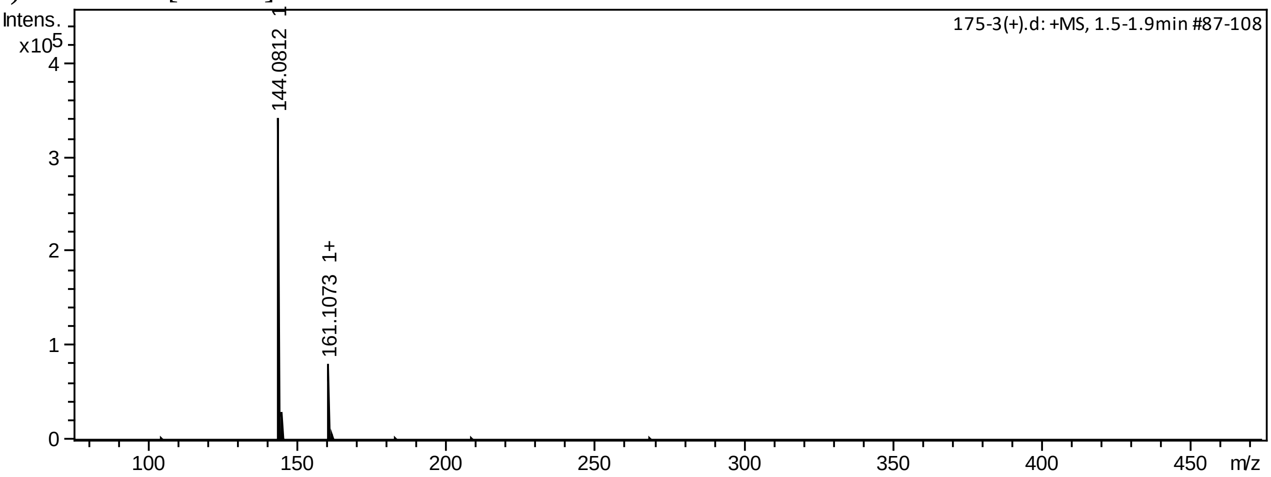

Figure S30. ^{1}H NMR (700.13 MHz, CD_3OD) spectrum of compound **5**

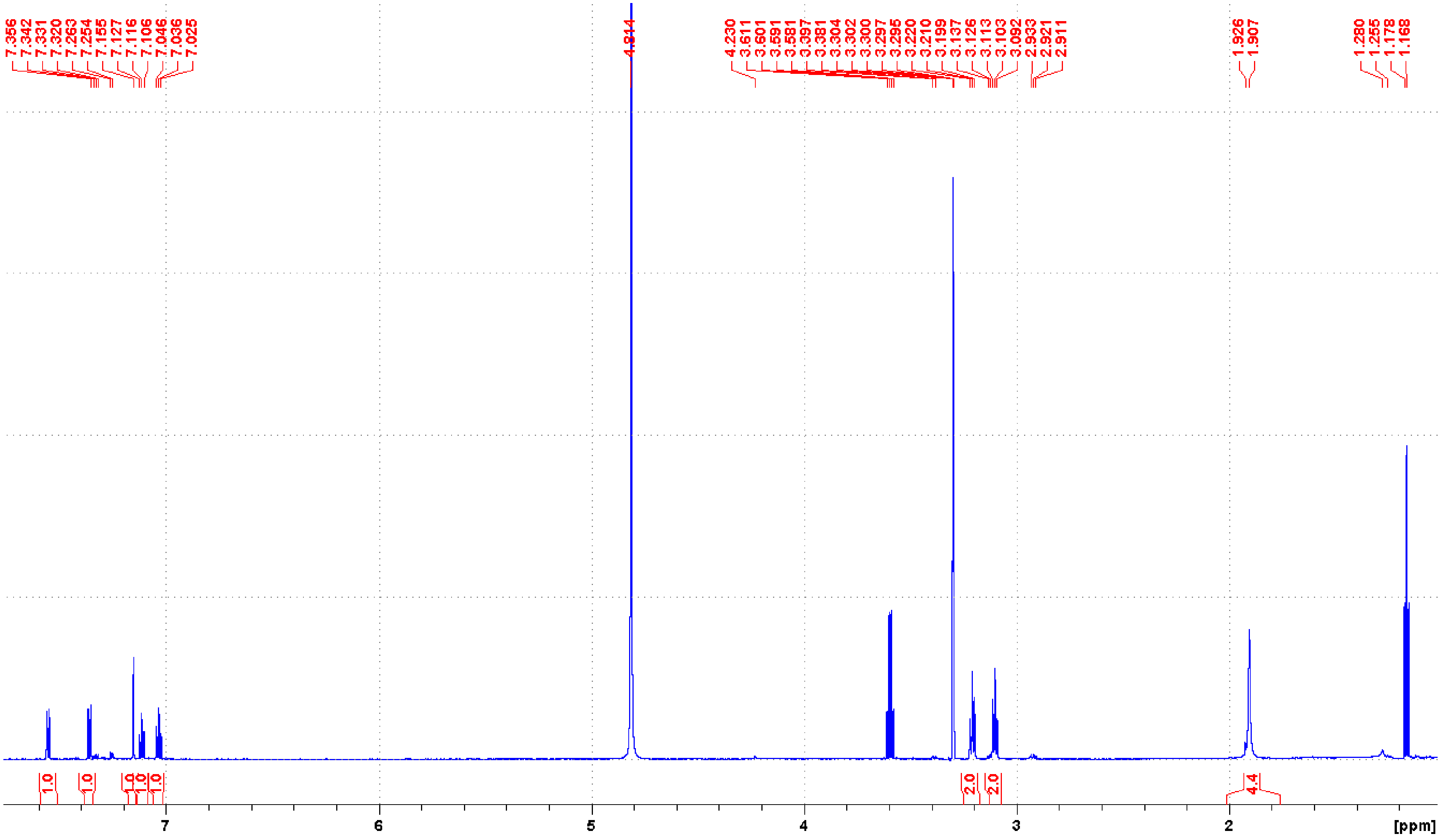

Figure S31. ^{13}C NMR (176.04 MHz, CD_3OD) spectrum of compound **5**

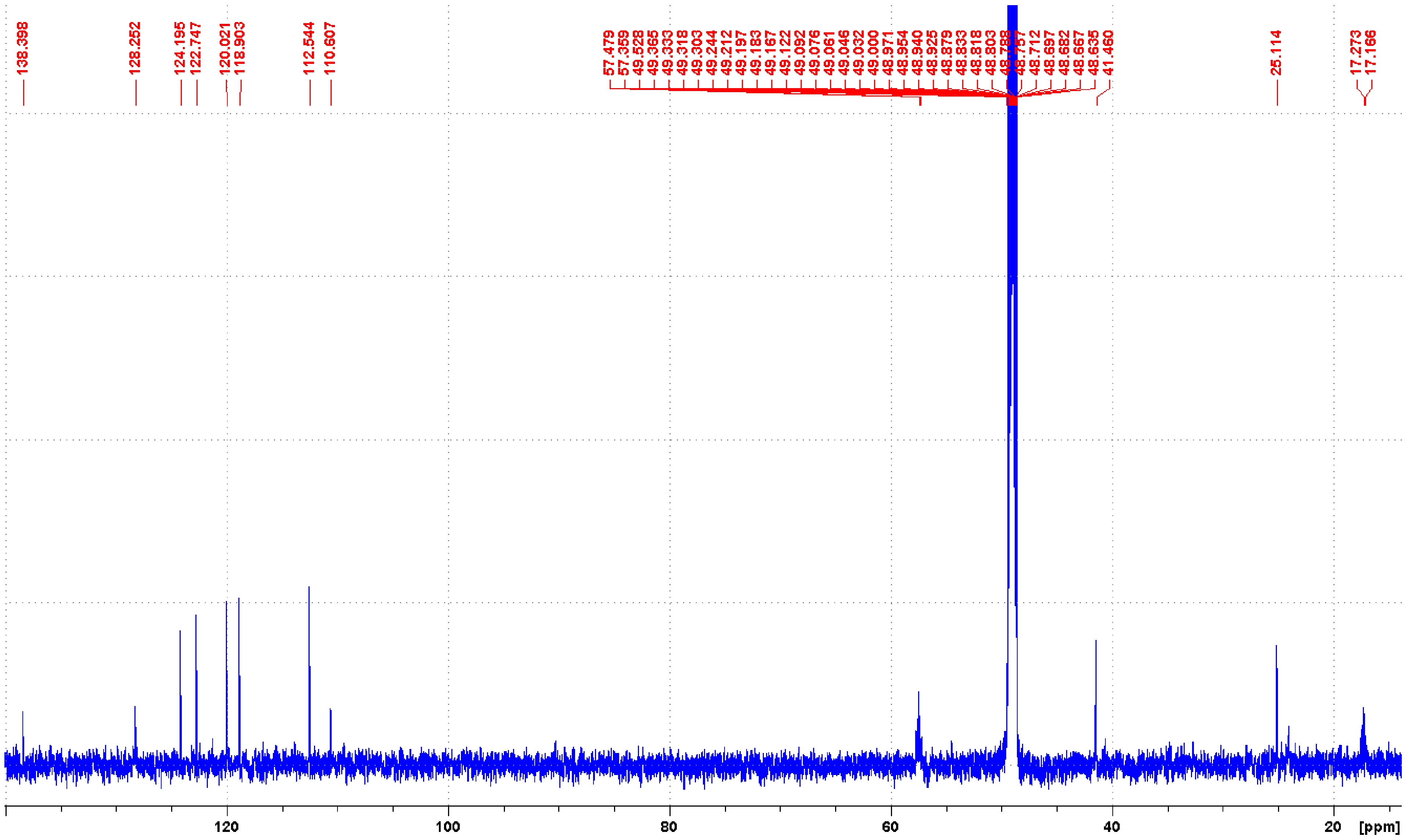

Figure S32. DEPT (176.04 MHz, CD_3OD) spectrum of compound **5**

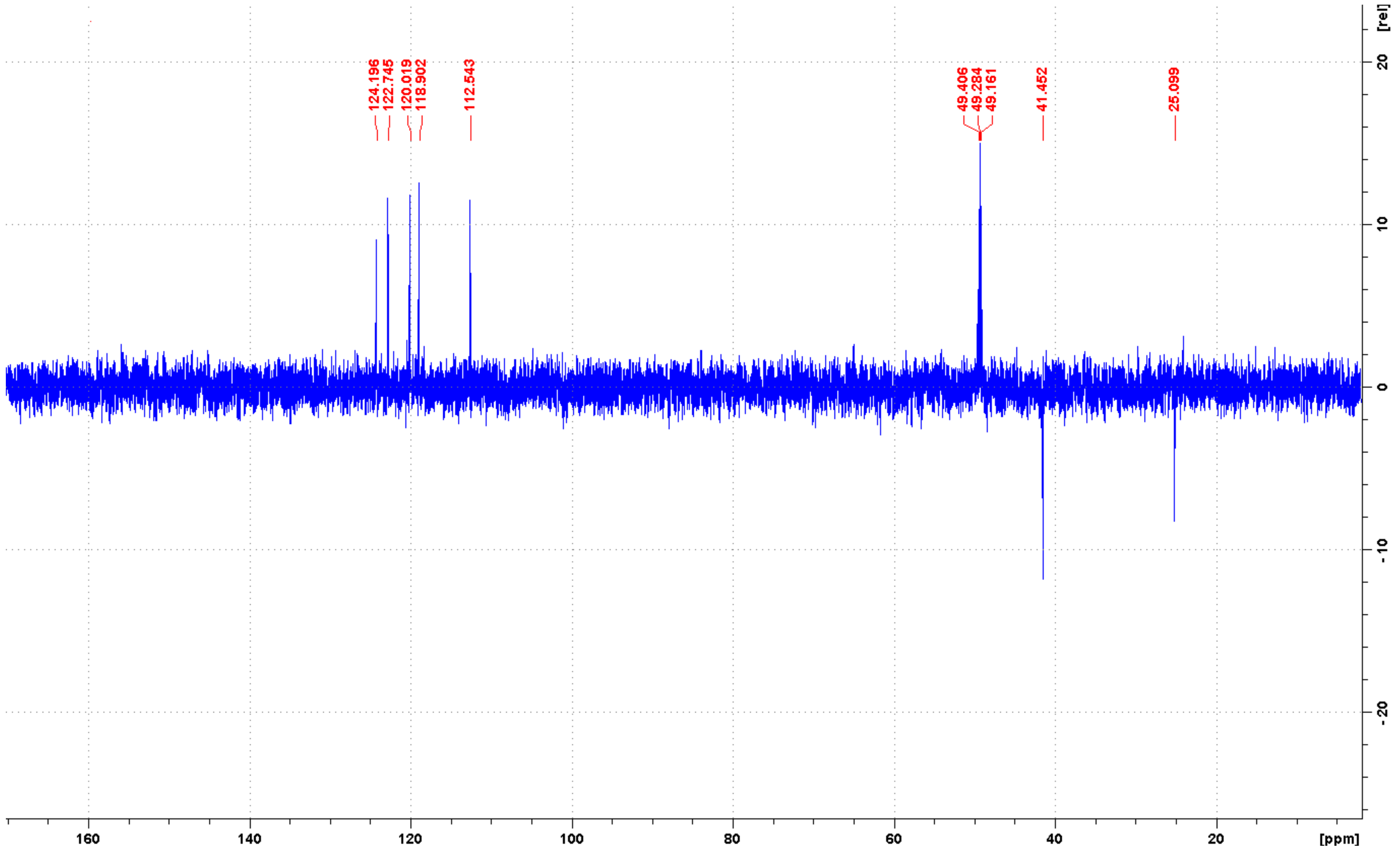

Figure S33. ^{1}H-^{1}H COSY (700.13 MHz, CD_3OD) spectrum of compound **5**

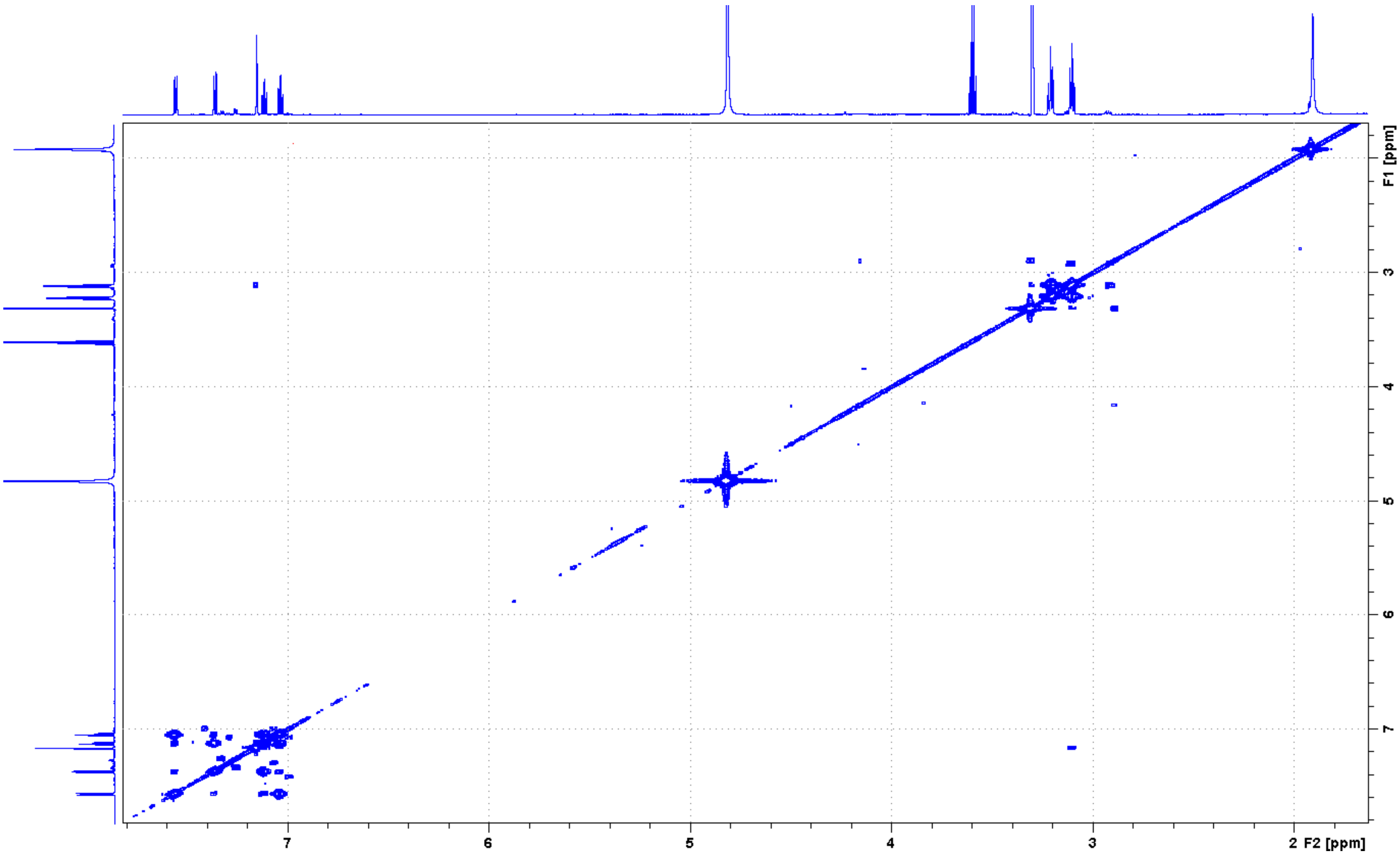

Figure S34. HSQC (700.13 MHz, CD_3OD) spectrum of compound **5**

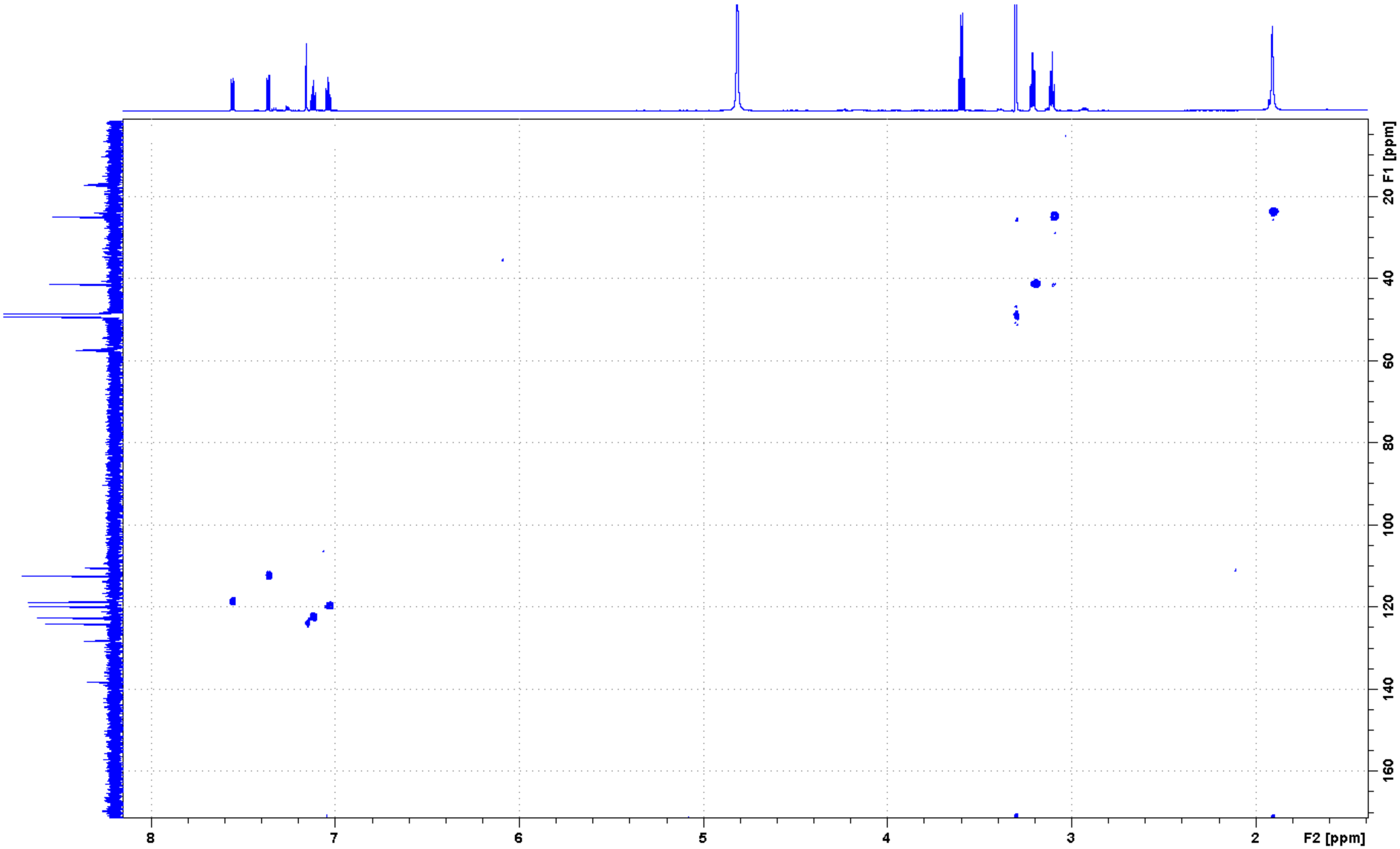

Figure S35. HMBC (700.13 MHz, CD_3OD) spectrum of compound **5**

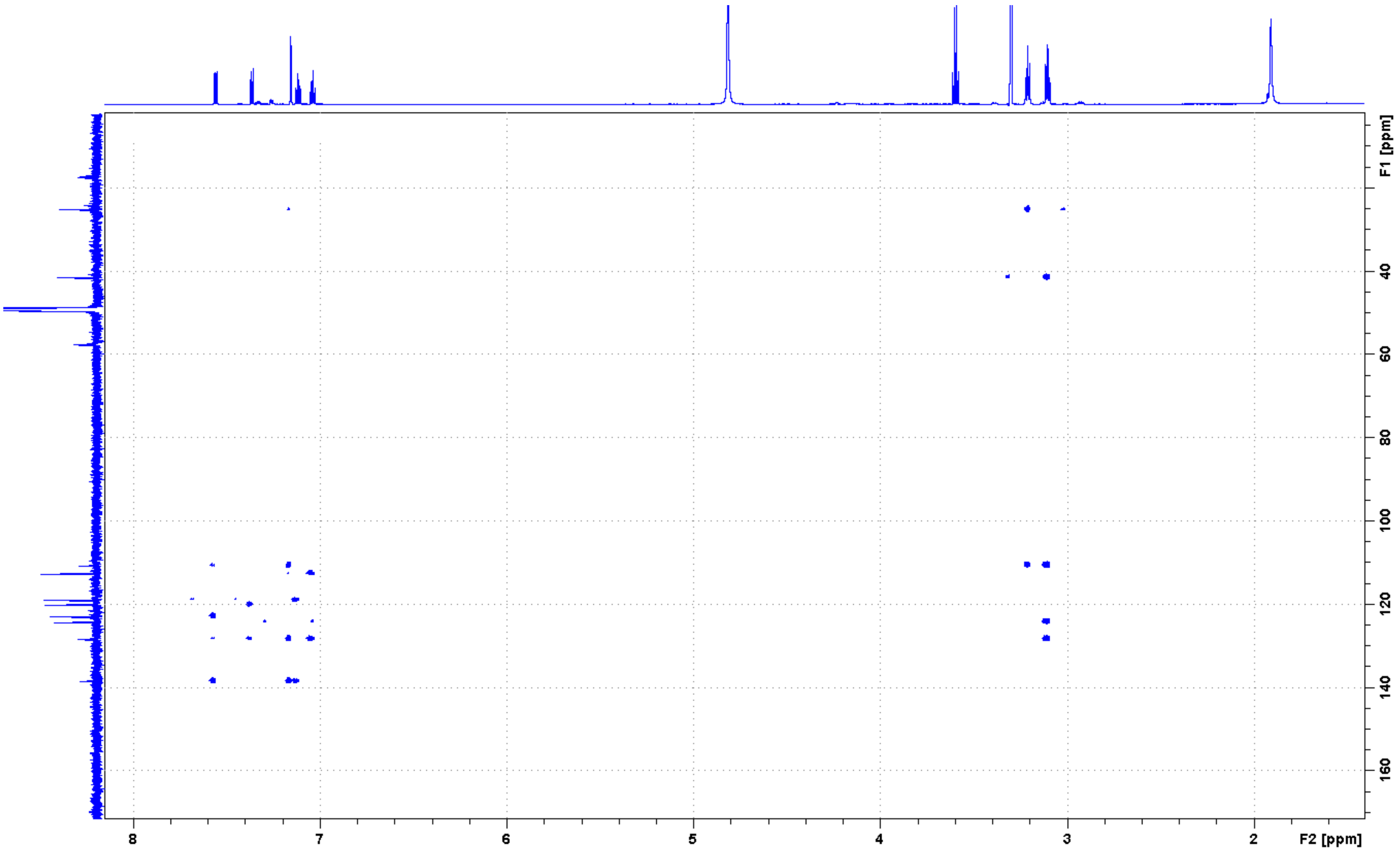

Figure S36. ROESY (700.13 MHz, CD_3OD) spectrum of compound **5**

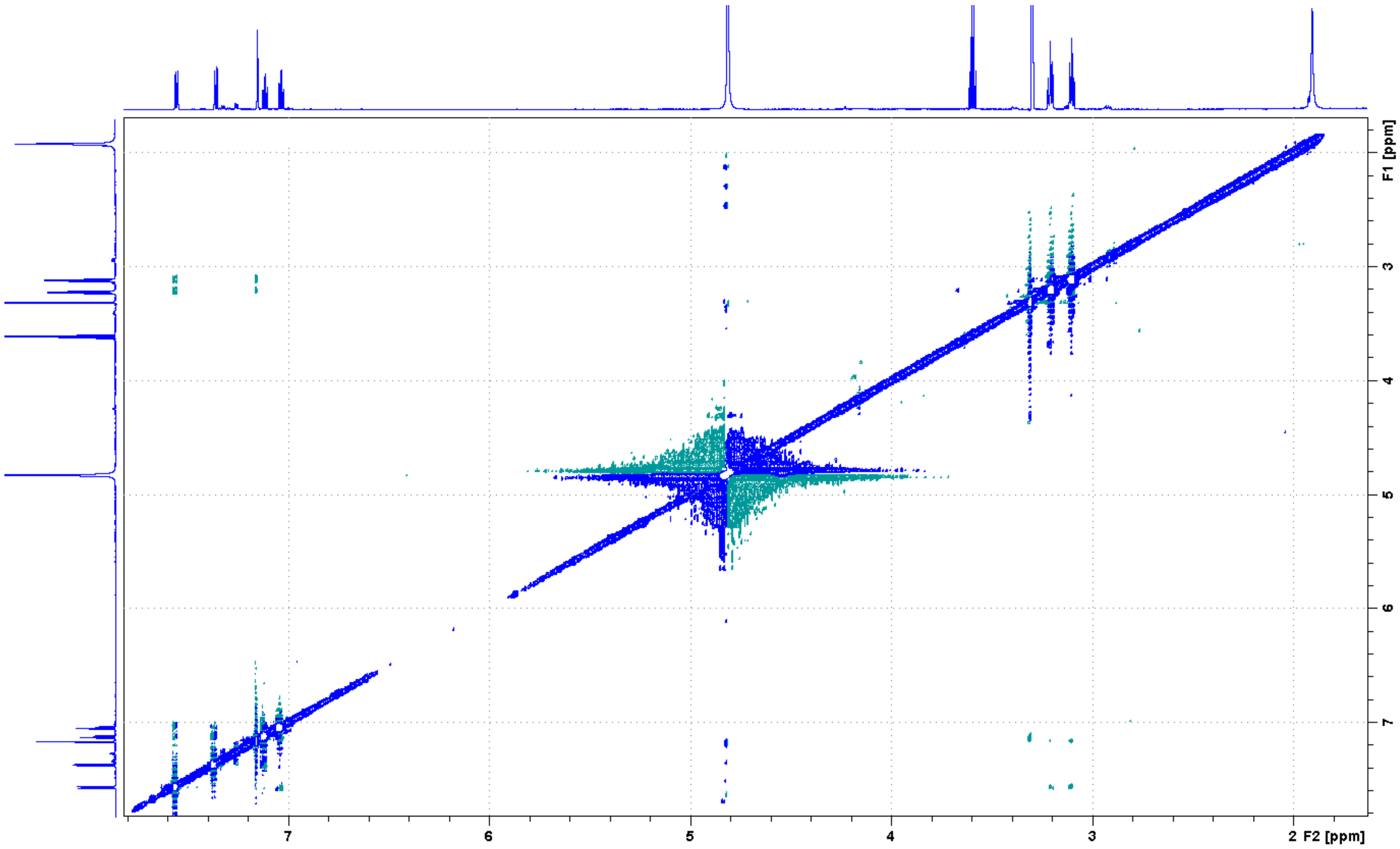

Supplement: Supplementary file 1 [file marinedrugs-22-00043-s001.zip › marinedrugs-2810878-supplementary.pdf]
